# Deregulation of selective autophagy during aging and pulmonary fibrosis: the role of TGFβ1

**DOI:** 10.1111/acel.12357

**Published:** 2015-06-09

**Authors:** Meredith L Sosulski, Rafael Gongora, Svitlana Danchuk, Chunmin Dong, Fayong Luo, Cecilia G Sanchez

**Affiliations:** Department of Medicine, Division of Pulmonary Diseases, Critical Care and Environmental Medicine, Tulane University Health Sciences CenterNew Orleans, LA, 70112, USA

**Keywords:** aging, autophagy, lung fibrosis, lung injury, mitophagy, PINK1, resveratrol, TGFβ1

## Abstract

Aging constitutes a significant risk factor for fibrosis, and idiopathic pulmonary fibrosis (IPF) is characteristically associated with advancing age. We propose that age-dependent defects in the quality of protein and cellular organelle catabolism may be causally related to pulmonary fibrosis. Our research found that autophagy diminished with corresponding elevated levels of oxidized proteins and lipofuscin in response to lung injury in old mice and middle-aged mice compared to younger animals. More importantly, older mice expose to lung injury are characterized by deficient autophagic response and reduced selective targeting of mitochondria for autophagy (mitophagy). Fibroblast to myofibroblast differentiation (FMD) is an important feature of pulmonary fibrosis in which the profibrotic cytokine TGFβ1 plays a pivotal role. Promotion of autophagy is necessary and sufficient to maintain normal lung fibroblasts’ fate. On the contrary, FMD mediated by TGFβ1 is characterized by reduced autophagy flux, altered mitophagy, and defects in mitochondrial function. In accord with these findings, PINK1 expression appeared to be reduced in fibrotic lung tissue from bleomycin and a TGFβ1-adenoviral model of lung fibrosis. PINK1 expression is also reduced in the aging murine lung and biopsies from IPF patients compared to controls. Furthermore, deficient PINK1 promotes a profibrotic environment. Collectively, this study indicates that an age-related decline in autophagy and mitophagy responses to lung injury may contribute to the promotion and/or perpetuation of pulmonary fibrosis. We propose that promotion of autophagy and mitochondrial quality control may offer an intervention against age-related fibrotic diseases.

## Introduction

While interstitial lung disease can affect both the young and the elderly, aging constitutes a significant risk factor for pulmonary fibrosis. Idiopathic pulmonary fibrosis (IPF) is an interstitial lung disease that is specifically associated with advancing age. An estimated 100 000 people in the United States have IPF, a chronic and lethal disorder with progressive decline of pulmonary function resulting in a median survival of only 3 years from time of diagnosis (Klingsberg *et al*., 2010). IPF is characterized by abnormal accumulation of myofibroblasts and formation of fibrotic foci in the lung, with excessive deposition of extracellular matrix components (Pardo & Selman, 2002).

Although the precise etiology of IPF remains elusive, significant advances in understanding this disease have been made through omics studies using lung biopsies from IPF patients (Korfei *et al*., 2011; Deng *et al*., 2013) Proteomic analysis has shown that an IPF-afflicted lung bears features of chronic cellular stress, with an increase in the unfolded protein response (UPR), accumulation of heat-shock proteins, and DNA damage (Korfei *et al*., 2011). It is also clear that TGFβ1 is an important factor in the pathogenesis of pulmonary fibrosis (Zhao *et al*., 2002). The predisposition for disrepair during aging coincides with increases in TGFβ1 signaling (Sueblinvong *et al*., 2012, 2014). TGFβ1 promotes fibroblast to myofibroblast differentiation (FMD) and myofibroblast survival by inducing a variety of signaling pathways, including the canonical TGFβ1 signaling pathway (Smad2/3-dependent) and noncanonical pathways, such as PI3K/AKT/mTOR pathway (Biernacka *et al*., 2011). The PI3K/AKT/mTOR pathway is constitutively activated in IPF fibroblasts within a polymerized collagen matrix (Nho & Hergert, 2014). The mTOR pathway regulates autophagy and insufficient autophagy has been shown to promote lung fibroblast differentiation (Araya *et al*., 2013).

Autophagy is a catabolic process conserved from yeast to mammals that provides a mechanism for stress recognition. Macroautophagy is the type of autophagy that involves sequestering portions of the cytosol (including soluble proteins, organelles, and aggregates) inside a forming autophagosomal membrane, which ultimately seals and fuses with lysosomes for degradation of the entrapped cargo. This process can occur in bulk or can be highly selective. The formation and elongation of the autophagosomal double membrane is controlled by the coordinated actions of autophagy-related genes (Atgs), including the microtubule-associated protein-1 light chain-3B (LC3B) (Klionsky *et al*., 2010). This process regulates several functions in the cell, including the reduction of oxidative stress primarily through mitochondrial turnover.

Under stress, mitochondrial dysfunction induces ubiquitin-dependent responses that involve remodeling of the mitochondrial proteome and organelle removal by mitophagy. PINK1, a serine/threonine kinase, is involved in the degradation of dysfunctional mitochondria, accumulates on depolarized mitochondria and recruits Parkin. At the depolarized mitochondria, Parkin, an E3 ubiquitin ligase, catalyzes the polyubiquitination of several substrates, triggering the engulfment of mitochondria by the autophagosome–lysosome pathway through recruitment of an LC3-binding protein, p62/SQSTM1 (Geisler *et al*., 2010).

Importantly, defects in mitochondrial turnover are associated with late-onset pathologies, the accumulation of defective mitochondria in aging tissues, and advanced oxidative stress (Bratic & Larsson, 2013; Schiavi & Ventura, 2014). Induction of mitochondrial turnover and recycling through autophagy is thus a legitimate pharmacological target in age-related lung diseases. Here, we demonstrate for the first time an age-related decline in autophagy and selective targeting of mitochondria for autophagic degradation, with reduced PINK1 expression, in animal models of pulmonary fibrosis. We propose that enhancement of autophagic flux and mitochondrial recycling by hormetic compounds can diminish the expression of damaging reactive oxygen species and help to maintain mitochondria function, the normal lung fibroblast phenotype and promotion of a healthy lung.

## Results

### Deficient autophagic response and increase in lipofuscin deposits are concomitant with disrepair in the aging lung

**Fig 1:**
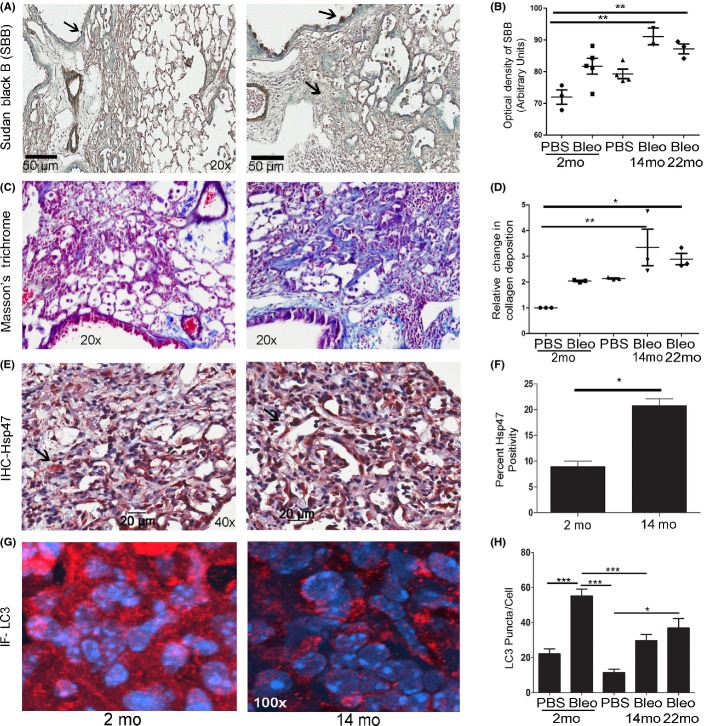
Bleomycin exposure exacerbates age-dependent differences in lipofuscin content, collagen deposition, Hsp47, and the autophagic marker LC3. Twenty-two-month-old (old) and 14-month-old (middle aged) mice exposed to bleomycin (Bleo), pulmonary fibrosis model, accumulates more lipofuscin as compared to 2-month-old (young) mice. (A) Representative images of Sudan Black B (SBB) staining for lipofuscin during fibrogenesis in young and middle-aged lung. Positive SBB appears dark brown–black. Nuclei were counterstained with methyl green (blue-green). Arrows show SBB-positive lipofuscin. (B) Quantification of SBB staining in 2-, 14-, and 22-month-old mouse lung sections. (C, D) Representative images and quantification of Masson’s trichrome staining to evaluate collagen deposition in young (shown), middle-aged (shown), and old lung. Positive collagen deposition appears blue. (E, F) Representative images and quantification of Hsp47 staining/cell demonstrate fibrogenesis in middle-aged lung. Positive cells stain red (NovaRed stain). Nuclei were counterstained with hematoxylin. (G, H) Representative images and quantification of LC3b staining show the autophagic response to injury in 2-, 14-, and 22-month-old mouse lung. Positive LC3b appears as red punctae. Nuclei were counterstained with DAPI (blue). *P < 0.05, **P < 0.01, ***P < 0.005.

The lungs of old mice show worse fibrosis after bleomycin-induced injury, an animal model that recapitulates the features of human pulmonary fibrosis, compared with the lungs from young mice (Sueblinvong *et al*., 2012; Peng *et al*., 2013). As shown, bleomycin promotes activation of TGFβ and AKT/mTOR pathways (Fig. S1). We studied bleomycin-exposed lungs from young mice (2 month old) and older mice, including 14 month old and 22 month old, for lipofuscin content, a nondegradable intralysosomal polymeric substance that accumulates during aging (Brunk & Terman, 2002). The purpose was to determine whether what have heretofore been considered age-related changes in lipofuscin can be detected and promoted in fibrotic lungs of young and late middle aged, as well as old mice, and whether these changes were progressive. Assessment of Sudan Black B staining and quantification by ImageJ revealed more lipofuscin aggregates in the lungs of 14-month-old mice versus 2-month-old mice after oropharyngeal aspiration of bleomycin (Fig.1A,B). No significant differences were detected between 14-month-old and 22-month-old mice. The results were confirmed using OxyIHC™ Oxidative Stress system for protein oxidation (Fig. S2A).

Collagen deposition was detected by Masson’s trichrome staining analysis. An increase in collagen deposition in the 14-month-old mice compared to young mice was observed (Fig.1C,D). Tissues were immunostained for heat-shock protein 47 (Hsp47), a collagen-binding glycoprotein localized in the endoplasmic reticulum, and a biomarker of early stages of fibrogenesis (Taguchi & Razzaque, 2007). Higher levels of Hsp47-positive cells (myofibroblast-type cells) appeared in 22- and 14-month-old mice compared to 2-month-old mice subjected to bleomycin (Fig.1E,F). After bleomycin exposure, an age-dependent increase in the expression of fibrotic markers, collagen type I (Col1), connective tissue growth factor (CTGF/CCN2), and plasminogen activator inhibitor-1 (PAI1) were confirmed by qRT–PCR (Fig. S2B). Compared to 2-month-old mice, the lungs of the older mice exposed to bleomycin exhibited severe interstitial and intra-alveolar pneumonia and fibrosis with the presence of myofibroblasts, as well as an increase in collagen fibers detected by electron microscopy images (Fig. S2C). No significant differences were detected between 14- and 22-month-old mice after lung injury. Interestingly, untreated 22-month-old mice have enlarged alveolar spaces compared to 2-month-old mice with higher levels of MMP9 expression (Fig. S2B).

The autophagic response to bleomycin was evaluated by microtubule-associated protein-1 light chain 3 β punctae (the lipidated form of LC3) immunostaining. The analysis revealed more punctae in younger mice (2 month old) when compared to older mice (14 and 22 month old) exposed to bleomycin (Fig.1G,H). Similarly, young mice had higher levels of LC3b punctae in both the interstitium and the respiratory epithelium (Fig. S3).

### Age-related changes in the mitophagic response to bleomycin

**Fig 2:**
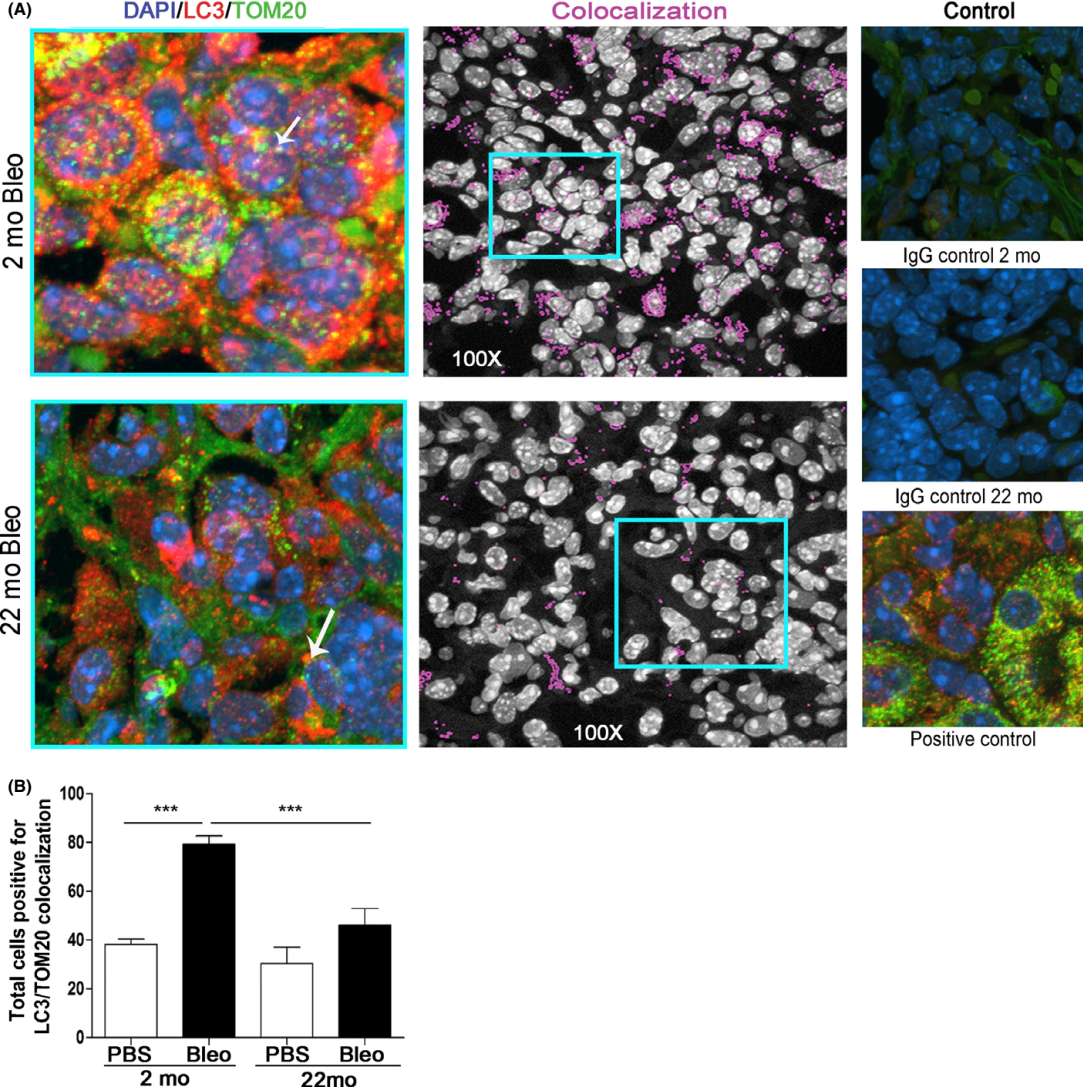
Deficient mitophagy response after lung injury in aged mice. (A) *Left*: Representative images of mitochondria inside autophagosomes (yellow), detected by colocalization of LC3b punctae (red) and TOM20 a mitochondria marker (green) in young (2 month old) and old (22 month old) bleomycin (Bleo) exposed mouse lung tissue. Nuclei were counterstained with DAPI (blue). *Center*: Colocalization labeled in pink on black and white images using the colocalization finder plug-in in Image J (NIH). *Right*: IgG-only controls in lung tissues used as negative controls. Kidney stained for LC3 and TOM20 used as positive control. (B) Quantification of number of mitochondria inside autophagic compartments in young and old mice exposed with vehicle (PBS) or bleomycin. ***P < 0.005.

In accordance with the increase in collagen deposition in aging lung (Fig. S4A) and mitochondrial dysfunction observed in age-related diseases (Sueblinvong *et al*., 2012; Bratic & Larsson, 2013), we chose to further evaluate changes in mitophagy in old mice (22 month old) and young mice (2-month-old) treated with bleomycin. Sequestration of mitochondria inside autophagic vacuoles was determined by colocalization of TOM20 (mitochondria) and LC3b (autophagosome) by immunofluorescence in lung tissue from control (PBS) and bleomycin-exposed young and old mice (Fig.2A,B). Quantification and analysis demonstrated that higher levels of mitophagy events are induced in the 2-month-old mice compared to older mice after bleomycin exposure (Fig.2B, Fig. S4B).

### TGFβ1 inhibits autophagy during myofibroblast differentiation in a time-dependent manner

**Fig 3:**
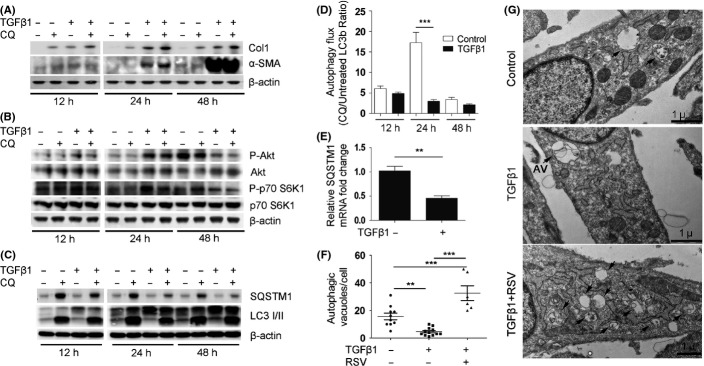
Repression of autophagy during FMD in lung fibroblasts by TGFβ1. TGFβ1 induces AKT/mTOR activation during the first 24 h of FMD, which occurs concomitantly with a decline in autophagy and expression of autophagy-related genes. (A) NHLF cultured for 12, 24, and 48 h with and without TGFβ1 and/or chloroquine (CQ) added 4 h prior to collection for Western blot (WB) analysis. Representative WB for fibrotic markers collagen type I (Col1), α-smooth muscle actin (α-SMA), and β-actin loading control. (B) Representative WB for mTOR pathway activation with the indicated antibodies and β-actin loading control. (C) Representative WB for autophagic markers with the indicated antibodies and β-actin loading control. Densitometry analysis of WB in Fig. S5A. (D) Quantification of LC3 punctae immunofluorescence indicates differences in the autophagic flux after 24-h treatment with TGFβ1. (E) Real-time RT–PCR (qRT-PCR) analysis for p62/SQSTM1 expression in TGFβ1-treated NHLF at 24 h. (F) Quantification of autophagic vacuoles (AV) in electron microscopy images in (G) performed on control (10 cells), TGFβ1 treated (12 cells), and TGFβ1 and RSV cotreatment (6 cells). (G) Confirmatory electron microscopy (TEM) for autophagosome analysis. Resveratrol (RSV) cotreatment is used as a tool to induce autophagosome formation. Arrows show autophagic vacuoles and are labeled AV. **P < 0.01, ***P < 0.005.

Due to the role of TGFβ1 in fibrogenesis, we tested the effect of TGFβ1 upon autophagy in NHLF by treating cells for 12, 24, and 48 h with 1 ng mL^−1^ of TGFβ1, alone or with 30 μm chloroquine (CQ) treatment 4 h prior to collection. The purpose was to observe time-dependent differences in the autophagy flux during the process of myofibroblast differentiation. The lysosome inhibitor CQ promoted accumulation of collagen type I (Col1) but not α-smooth muscle actin (α-SMA), even in the absence of TGFβ1, suggesting that in normal human lung fibroblasts, as well as differentiated myofibroblasts, Col1 is targeted for lysosomal degradation, but not α-SMA (Fig.3A). We next evaluated the time-dependent regulation of mTOR during the process of myofibroblast differentiation after TGFβ1 induction. Western blots using antibodies specific for the active (phosphorylated) forms of AKT and a downstream target of mTOR, p70 S6-Kinase 1, indicated that TGFβ1 induced the AKT/mTOR pathway in a time-dependent manner with an activation peak at 24 h (Fig.3B).

Western blots also confirmed that TGFβ1 reduced autophagy at 24 h (determined as the increase in LC3II levels upon addition of CQ), but no significant differences were noticed after 48 h (Fig.3C, Fig. S5A). Changes in the autophagy flux were confirmed by an increase in the immunofluorescence of the LC3b punctae upon CQ treatment (Fig.3D, S5B). Analysis of the CQ treatment confirms that the maximum inhibition of the autophagy flux (CQ treated/untreated) corresponds temporally with the peak in mTOR activity (Fig.3D).

Importantly, p62/SQSTM1 protein levels assessed by Western blot declined in TGFβ1-treated fibroblasts and CQ treatment failed to increase p62/SQSTM1 to the same levels observed in control fibroblasts (Fig.3C). The measurement of p62/SQSTM1 expression as a marker of autophagic flux is controversial and misinterpreted; this protein is subject to regulation at both transcriptional and post-translational levels. Analyses by qRT–PCR showed that TGFβ1 repressed p62/SQSTM1 gene expression by 50% (Fig.3E), which indicates that p62/SQSTM1 cannot be used as an autophagy flux marker in this context.

Finally, we confirmed the inhibitory effects of TGFβ1 on autophagy in the human lung fibroblasts by semiquantitative analysis of number of autophagic vacuoles (AV) detected by electron microcopy, as presented in Fig.3F,G.

### TGFβ1 modulates the transcription of autophagy-related genes during myofibroblast differentiation

**Fig 4:**
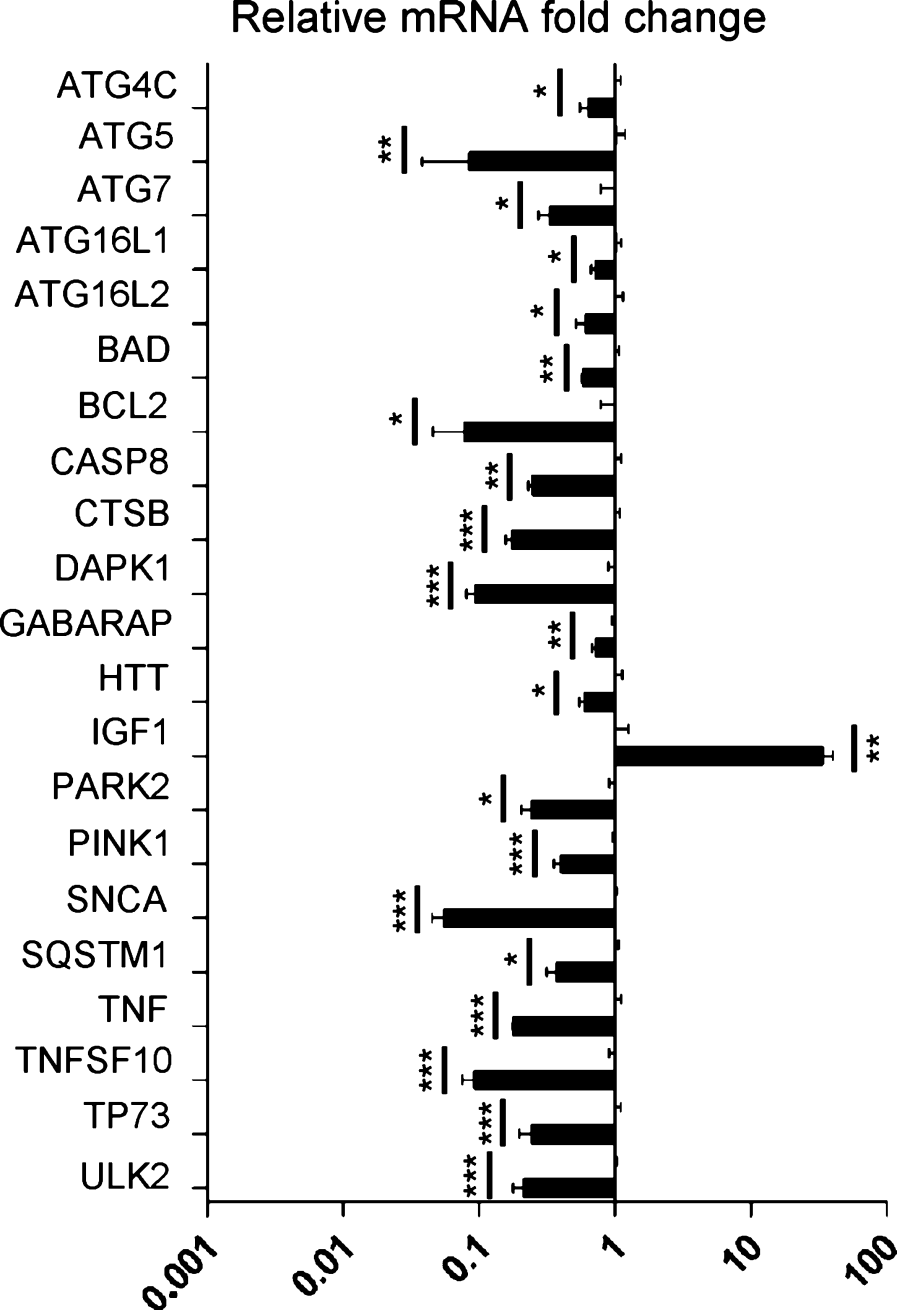
TGFβ1 modulates the transcriptional level of major autophagy regulator genes during myofibroblast differentiation. Real-time RT–PCR analysis with independent primers for indicated genes to confirm significant fold changes detected in expression. A base-10 log scale is used for the *x*-axis of graph. *P < 0.05, **P < 0.01, ***P < 0.005.

Our findings regarding TGFβ1-dependent repression of p62/SQSTM1 mRNA levels prompted us to determine whether other autophagy-related genes were regulated similarly. RNAs from NHLF untreated and treated with TGFβ1 for 24 h were analyzed by qRT–PCR in an array format for 84 key genes involved in autophagy as components of the molecular machinery and regulators. The results showed that 21 different autophagy-related mRNAs were significantly regulated by TGFβ1 in NHLF (Fig. S6). These results were confirmed by qRT–PCR with an independent set of primers (Fig.4, Supporting Information). The complete list of gene expression changes are shown in the Table S1. Interestingly, TGFβ1 repressed genes involved in autophagosome formation, maturation, coregulators of autophagy/apoptosis, and regulators of autophagy in response to intracellular pathogens and other intracellular signals (Fig.4). Only one gene, IGF1, was significantly upregulated (>30-fold change) by TGFβ1 (Fig.4).

### TGFβ1 modulates PINK1 expression, mitophagy, and mitochondrial homeostasis during fibrogenesis

**Fig 5:**
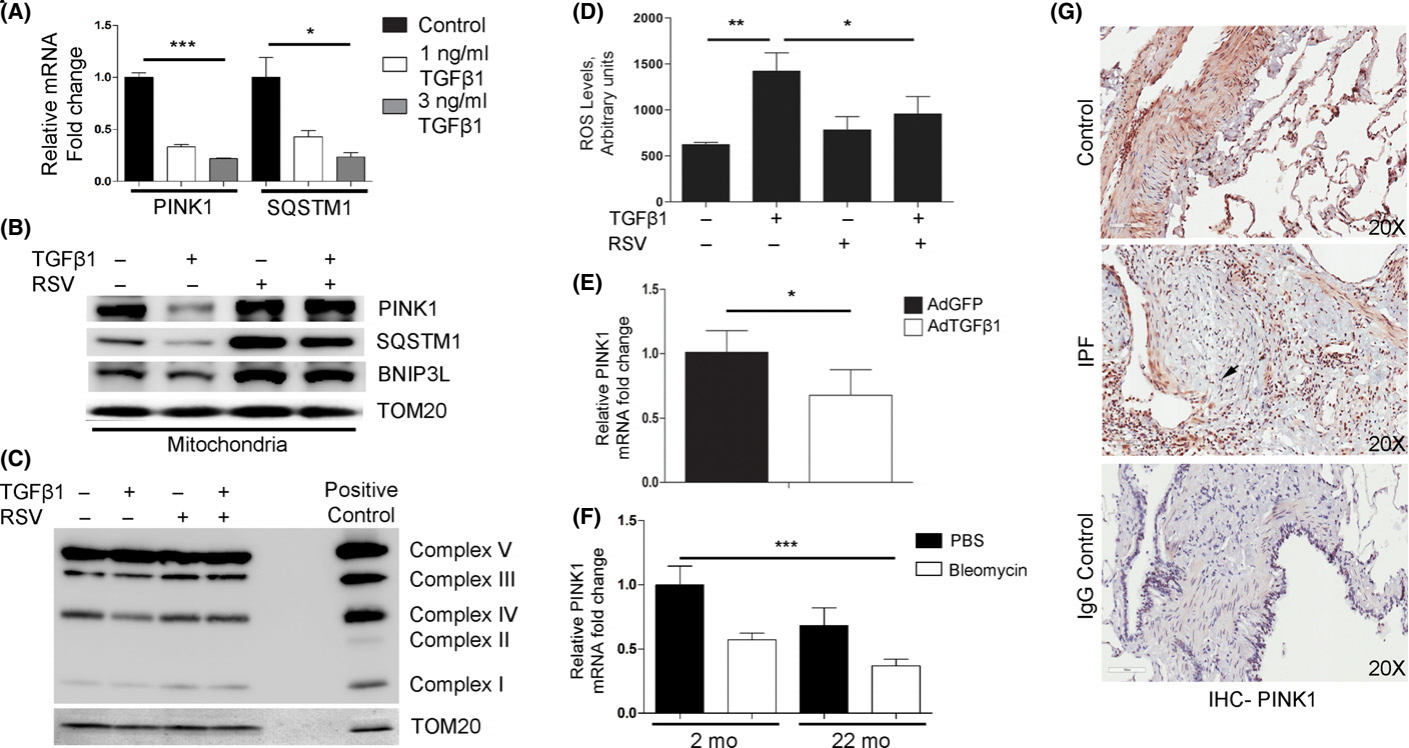
Deregulated mitochondrial homeostasis and PINK1 expression in pulmonary fibrosis. TGFβ1 treatment alters expression of mitophagy-related proteins, specifically PINK1, consistent with IPF-related changes. (A) qRT–PCR analysis for TGFβ1 dose-dependent changes in the transcriptional levels of PINK1 and p62/SQSTM1 in NHLF after 24 h. (B) Representative WB of mitochondria isolated from NHLF treated or untreated with TGFβ1 and/or resveratrol (RSV) probed for indicated antibodies. TOM20 was used as loading control. (C) Representative WB for OXPHOS complexes from NHLF treated or untreated with TGFβ1 and/or RSV. TOM20 was used as loading control. (D) Quantification of reactive oxygen species from NHLF treated with or without TGFβ1 and/or RSV. E) qRT-PCR analysis for mRNA expression levels of PINK1, at 7 days post oropharyngeal aspiration of control adenovirus-GFP (AdGFP) or adenovirus-TGFβ1 (AdTGFβ1) in mice (*n* = 5 per treatment). (F) qRT–PCR analysis for PINK1 expression in 2-month-old (*n* = 5) and 22-month-old mice (n = 5) after oropharyngeal aspiration of bleomycin (Bleo) or PBS vehicle only, at 14 days postexposure. (G) IHC in lung tissue samples from an IPF patient and a control patient show differential expression of PINK1. Positive cells appear red (NovaRed stain). Nuclei counterstained with hematoxylin appear blue. Arrow shows fibrotic lesion. IgG only was used as negative control. *P < 0.05, **P < 0.01, ***P < 0.005.

qRT–PCR analysis confirmed TGFβ1-induced transcriptional inhibition for p62/SQSTM1 and PINK1 in dose-dependent manner (Fig.5A). These results suggest that deficient mitochondrial targeting for mitophagy could occur during FMD through TGFβ1-induced deregulation of the PINK1/PARKIN/p62 pathway.

To determine whether the reduction in PINK1 and p62/SQSTM1 expression observed during the process of FMD is reflected in the level of recruitment of mitochondria for degradation, we performed total protein analysis and mitochondria isolation in NHLF treated with or without TGFβ1 and/or resveratrol (RSV), a hormetic compound that promotes autophagy and inhibits FMD. Western blot analysis demonstrated that the TGFβ1-mediated decline in p62/SQSTM1 in total cell protein was also observed in the isolated mitochondria, and mitochondrial protein was normalized with TOM20 (Fig.5B). Similarly, the levels of PINK1 and BNIP3L (Nix), a receptor for mitophagy, declined in the isolated mitochondria from TGFβ1-treated cells. (Fig.5B). On the contrary, cotreatment with RSV maintains the expression levels of PINK1, p62/SQSTM1, and BNIP3L in mitochondria (Fig.5B). Defects in mitochondria recycling were confirmed using NHLF with and without 4-h CQ treatment. CQ increased the accumulation of PINK1 and TOM20 as a result of the active autophagy flux in NHLF; however, this process was impaired in the presence of TGFβ1. By contrast, RSV promoted active mitophagy (Fig. S7).

To demonstrate the functional relevance of these results, we performed Western blot analyses of the relative levels of the oxidative phosphorylation (OXPHOS) complexes in mitochondrial preparations from these cells. Levels of complex III and IV declined in mitochondria from TGFβ1-treated cells, which were reversed by cotreatment with RSV (Fig.5C). Assessment of ROS levels using the DCFH-DA assay demonstrated an increase in ROS consequent to TGFβ1 that was reversed by RSV treatment (Fig.5D).

To demonstrate whether the transcriptional downregulation of PINK1 by TGFβ1 in normal human lung fibroblasts is recapitulated in animal models of pulmonary fibrosis, we exposed mice to 3 × 10^8^ PFU of replication-deficient adenovirus encoding either GFP (AdGFP) or active TGFβ1 (AdTGFβ1). Real-time RT–PCR and Western blot analyses from whole lung extracts were performed 7 days postinfection. The results demonstrated a TGFβ1-associated reduction in PINK1 in the lung 7 days postinfection (Fig.5E). A corresponding reduction in PINK1 protein levels was confirmed by Western blots and densitometry (Fig. S8A,B). We obtain similar results using the bleomycin animal model of pulmonary fibrosis (Fig.5F). PINK1 expression was found to be reduced in the 22-month-old mice compared to 2-month-old mice. Finally, immunohistochemistry performed on human IPF lung and control lung samples confirmed low levels of PINK1 in the IPF tissue and nondetectable PINK1 staining in the fibrotic foci (Fig.5G).

### Fibroblasts undergoing active autophagy resist the remodeling effects of TGFβ1

**Fig 6:**
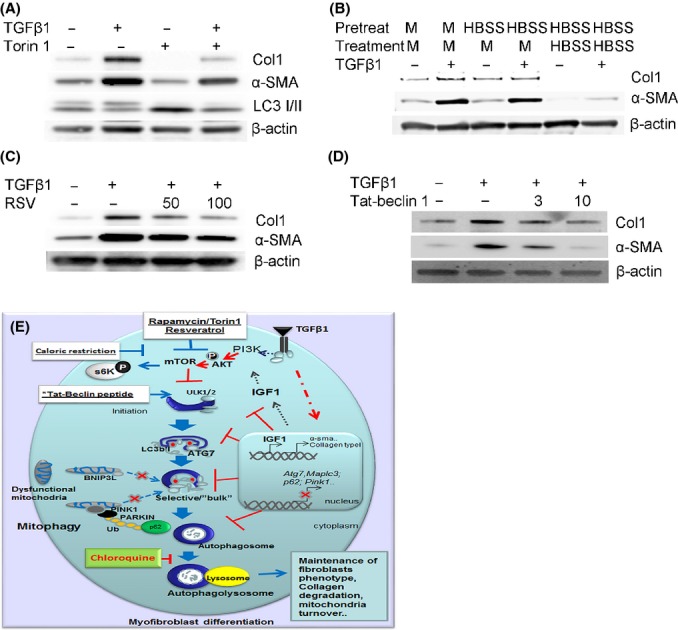
Fibroblasts undergoing active autophagy resist the remodeling effects of TGFβ1. Inhibition of FMD is demonstrated by Western blot from total extracts derived from NHLF treated with TGFβ1 and autophagy inducers. Representative WB shown was probed with antibodies to fibrotic markers collagen type I (Col1) and α-smooth muscle actin (α-SMA) using β-actin as protein loading control. (A) NHLF cultured in the presence of TGFβ1 and Torin 1 for 24 h. (B) NHLF precultured in nutrient-restricted conditions (HBSS) or complete media (M) then cotreated with TGFβ1 for an additional 24 h. (C) TGFβ1 alone or cotreatment with TGFβ1 plus resveratrol (RSV) at multiple doses for 24 h. (D) NHLFs were cultured in the presence of TGFβ1 and doses of Tat-beclin 1 peptide for 24 h. (E) Proposed model for TGFβ1 repression of autophagy during myofibroblast differentiation. TGFβ1 activates the PI3K/AKT/mTOR pathway which inhibits the initiation of the autophagosome formation. TGFβ1 will impart, directly or indirectly, deregulation of autophagy-related genes that will inhibit autophagy at different levels of the process, including autophagosome formation, selectivity, and degradation. Furthermore, TGFβ1 inhibits the expression and recruitment of PINK1/PARKIN/p62 to the mitochondria, promoting accumulation of dysfunctional mitochondria. Fibroblasts, as well as myofibroblasts, use autophagy as a means to control the levels of intracellular Col1. Deficient autophagy promotes Col1 and Hsp47 accumulation. Induction of autophagy in an mTOR-dependent (Rapamycin, Torin 1, caloric restriction or RSV) or mTOR-independent manner (LiCl, Tat-beclin 1) inhibits FMD. On the contrary, inhibition of autophagy promotes myofibroblast differentiation independently of TGFβ1.

Myofibroblasts are key players in pulmonary fibrosis and the major source of Hsp47 expression and interstitial collagen deposition. We explored possible therapeutic implications of autophagy induction relative to FMD in cell culture. NHLFs were treated with TGFb1 in presence of autophagy inducers. The FMD response to TGFβ1 was evaluated by Western blot for Col1 and α-SMA. The results indicates that selective inhibition of mTOR with Torin-1 repressed TGFβ1-mediated induction of Col1 and α-SMA, while increasing the LC3b-II/LC3b-I ratio (Fig.6A, Fig. S9A). Calorie restriction decelerates mTOR-driven aging in cells as well as in organisms, including humans (Blagosklonny, 2010). NHLFs were pretreated with complete media or HBSS media to induce nutrient restriction for 24 h followed by treatment with TGFβ1 for an additional 24 h. The results from Western blots (and qRT–PCR, data not shown) demonstrated that preconditioning with nutrient restriction promoted resistance to induction of fibrotic markers, Col1 expression, and α-SMA, by TGFβ1 (Fig.6B, Fig. S9B). Nonspecific hormetic compounds such as RSV promoted autophagy and also prevented FMD in a dose-dependent manner (Fig.6C, Fig. S9C).

To more directly implicate autophagy in repression of TGFβ1-mediated FMD, we used Tat-beclin 1, a membrane permeable peptide that selectively induces autophagy (Shoji-Kawata *et al*., 2013). Treatment with Tat-beclin 1 peptide reduced the expression of fibrotic markers in a dose-dependent manner (Fig.6D, Fig. S9D). Moreover, using a genetic approach, we confirmed that inhibition of autophagy by knockdown of ATG5 and ATG7 in NHLF promoted expression of α-SMA and Col1 (Fig. S10A,B). Taken together, these results indicate that active autophagy prevents FMD and that promotion of autophagy may be necessary and sufficient to maintain normal lung fibroblasts’ fate.

## Discussion

Our data support the role of autophagy and mitophagy, as protective mechanisms, against pulmonary fibrosis. In accord, our studies in animal models of pulmonary fibrosis demonstrated that susceptibility to pulmonary fibrosis during aging correlates with reduced autophagy, measured by the number of autophagosomes and mitochondria associated with autophagosomes, an increase in lipofuscin deposits and age-dependent decline in PINK1 expression. Also, lung tissues from IPF patients express reduced levels of PINK1. Furthermore, we found that TGFβ1 represses autophagy, mitochondrial recycling, and homeostasis in normal human lung fibroblasts during FMD and inhibits PINK1 expression during pulmonary fibrosis. Autophagy is a stress response and a quality control mechanism that protects against cellular stress and injury (Aguirre *et al*., 2014; Chang *et al*., 2015). Thus, impaired autophagic activity and mitophagy in response to injury may contribute to the onset of age-related lung diseases.

Our data identified an inverse correlation between the number of autophagosomes and the accumulation of lipofuscin and collagen deposits in bleomycin-treated mice. We propose that deficient autophagy can exacerbate lung injury by promoting oxidative stress, dysfunctional mitochondria, and lipofuscin deposits. Moreover, the accumulation of lipofuscin further limits autophagic turnover, exacerbating fibrosis. Our finding that repressed autophagic response during aging contributes to lung fibrosis contrasts with the excessive autophagy that underlies the pathogenesis of emphysema (Chen *et al*., 2008). Our results support the possibility that autophagy may be a critical determinant of the response to lung injury by settling the development of fibrosis or emphysema.

Previous studies using lung biopsies from IPF patients reported a diminution in autophagy (Patel *et al*., 2012; Araya *et al*., 2013). Herein, we impart an appreciation for TGFβ1-mediated regulation of the autophagic response during lung aging as part of the normal and pathological response to injury and fibrogenesis. Our findings demonstrate that autophagy may restrain transdifferentiation of normal human lung fibroblasts. A proposed model of these events is shown in Fig.6E. TGFβ1 reduces autophagy, in part, by selectively repressing expression of autophagy mediators in normal lung fibroblasts. Furthermore, TGFβ1 reduced autophagy flux, PINK1/p62-dependent mitochondrial recycling, and mitochondrial oxidative phosphorylation. This study connects autophagy and mitochondrial homeostasis to cell fate during fibrogenesis. Nevertheless, a cell type-specific genetic approach and the redundancy of mechanisms that regulate mitochondrial homeostasis need to be investigated, as pulmonary fibrosis is a complex disease that involves multiple interacting signaling pathways (Hecker *et al*., 2014).

We found that TGFβ1 stimulates the expression of the cytokine IGF1, an inhibitor of autophagy and a marker of aging in healthy adults (Vestergaard *et al*., 2014). In some organisms, mutations that reduce the activity of the IGF1/AKT pathway increase longevity (Feng & Levine, 2010). Like TGFβ1, IGF1 is elevated in the lungs of patients with IPF as well as in animal models of pulmonary fibrosis (Honeyman *et al*., 2013). IGF1 and TGFβ1 can act synergistically to promote changes in cell metabolism, survival, and cytoskeletal reorganization (Honeyman *et al*., 2013).

As shown in our studies, TGFβ1 reduces the expression of p62/SQSTM1. p62/SQSTM1 serves as multifunctional regulator of cell signaling involved in selective autophagy, intracellular trafficking and the NRF2 antioxidant response (Ichimura *et al*., 2013; Calderilla-Barbosa *et al*., 2014). Relative to the latter activity, defects in p62/SQSTM1 may contribute to the deregulation in NRF2 activity seen in myofibroblasts and pulmonary fibrosis (Ichimura *et al*., 2013; Hecker *et al*., 2014). Thus, p62/SQSTM1 could provide dual protection to stressed cells by facilitating both autophagy and the Nrf2-mediated antioxidant response. The relevance of p62/SQSTM1 function to pulmonary fibrosis is further supported by studies describing accelerated aging and age-related pathologies associated with loss of p62/SQSTM1 (Bitto *et al*., 2014).

Our findings indicate PINK1 is another target repressed by TGFβ1. Studies demonstrating that TGFβ1 regulates phosphatase and tensin homolog (PTEN), a driver of PINK1 expression, are consistent with this finding (Chow *et al*., 2008). PTEN deficiency promotes fibrogenesis (He *et al*., 2014; Roe *et al*., 2015). The relevance of our findings was further confirmed by our recent observation that PINK1 knockout mice have a pro-inflammatory environment characterized by increased levels of IL-6 (data not shown). In fact, recent studies in PINK1 null mice demonstrated high levels of TGFβ1 and susceptibility to pulmonary fibrosis (Knight *et al*., 2003; Bueno *et al*., 2015). The TGFβ1–PINK1 interaction could constitute a feed forward cycle that favors the perpetuation of fibrosis that is characteristic of the IPF lung. Further analyses of PINK1 knockout mice will better define the role of PINK1 in lung aging and pulmonary fibrosis.

It is still unclear whether or how TGFβ1 influences human aging. In *C. elegans*, the TGFβ signaling pathway represses lifespan (Hirose *et al*., 2003). In humans, a correlation between a polymorphism in the TGFβ1 gene and longevity suggests a similar function (Carrieri *et al*., 2004). The aging lung displays a profibrotic phenotype characterized by enhanced TGFβ1 expression and signaling (Doyle *et al*., 2010; van der Kraan *et al*., 2012; Sueblinvong *et al*., 2012). Our data describe the influence of TGFβ1 upon aging through changes in autophagy, mitochondrial homeostasis, and promotion of aberrant responses to lung injury.

At the cellular level, reduction of autophagy and mitophagy could abet myofibroblast differentiation and assist adaptation to metabolic changes and thereby prevent apoptosis (ML Sosulski, R Gongora, CG Sanchez unpublished data). However, deregulated cellular proteostasis and mitochondria recycling may contribute to other features of interstitial lung diseases, such as disrupted cellular redox, chronic inflammation, and increased vulnerability of the lung epithelia to second-hit injury (Hecker *et al*., 2014; Hawkins *et al*., 2015). Conversely, moderate induction of autophagy promotes resistance to oxidative stress and extension of lifespan (Pyo *et al*., 2013). In fact, we and others found that hormetic compounds like resveratrol promote autophagy and mitochondrial homeostasis, while inhibiting FMD and pulmonary fibrosis in animal models (Sener *et al*., 2007; Akgedik *et al*., 2012). Finally, we propose promoting autophagy and mitochondrial homeostasis to intervene against age-related lung diseases like pulmonary fibrosis.

## Experimental procedures

### Cell culture, reagents, and transfection

Normal human lung fibroblasts (NHLFs) were obtained from ATCC and maintained in Fibroblast Growth Medium-2 (FGM-2, Lonza, Walkersville, MD, USA) before serum starving cells in Fibroblast Basal Medium (FBM, Lonza) supplemented with 0.2% bovine serum albumin (BSA, Gemini Bio-Products Inc., Woodland, CA, USA). During treatments, NHLFs were cultured in FBM plus 0.2% BSA. For siRNA knockdown experiments, cells were transfected using the Neon Transfection System (Invitrogen, Carlsbad, CA, USA). Additional information is provided in Supplemental Experimental Procedures.

### Mice & tissue samples

All animal protocols were performed as approved by the Tulane University Institutional Animal Care and Use Committee. C57BL/6 male mice were obtained from the Jackson Laboratory (Bar Harbor, ME, USA) and housed in sterile conditions. Additional details are provided in Supplemental Experimental Procedures.

### Western blots

Cells were harvested in 1× RIPA Buffer (Cell Signaling, Danvers, MA, USA), sonicated, and quantified using the Bradford Method (Bio-Rad Laboratories Inc., Hercules, CA, USA). Flash-frozen lung tissue from mice was homogenized in 1× RIPA Buffer with an EDTA-free protease inhibitor cocktail (Roche Applied Science, Indianapolis, IN, USA). Additional details are provided in Supplemental Experimental Procedures.

### Antibodies

Antibody details are provided in Supplemental Experimental Procedures.

### Electron microscopy

Information is given in Supplemental Experimental Procedures.

### Histology, immunofluorescence–paraffin staining, and immunohistochemistry

Histologic sections of lung tissue (4 μm) were deparaffinized and rehydrated according to the standard protocol. Additional details are given in Supplemental Experimental Procedures.

### Sudan black B staining

Tissue slides were deparaffinized and rehydrated to 70% ethanol. Freshly prepared Sudan Black B (SBB; Fisher Scientific, Pittsburgh, PA, USA) stain was prepared as previously described (Georgakopoulou *et al*., 2013), and slides were incubated in SBB for 20 min. Nuclei were counterstained with methyl green.

### Oxidative stress assays

In cells, ROS levels were measured by detection of DCF, the fluorescent product formed from the oxidation of 2,7-dichlorodihydrofluorescein diacetate (DCFH-DA, Sigma, St. Louis, MO, USA). See Supplemental Experimental Procedures.

### Imaging & quantitative analysis

Information is given in Supplemental Experimental Procedures.

### RNA isolation, qRT–PCR, and gene expression array

Total RNA was isolated using Trizol® Reagent (Invitrogen) according to manufacturer’s instructions. The gene expression profile was evaluated using RT^2^ Profiler™ PCR array for autophagy (PAHS-084A; SABiosciences, Frederick, MD, USA) according to standard protocol. Details are given in the Supplemental Experimental Procedures.

### Mitochondria isolation

Mitochondria were isolated from NHLF post-treatment using the Thermo Scientific (Rockford, IL, USA) mitochondrial isolation kit for cultured cells according to the manufacturer’s protocol.

### Statistical analysis

All data are expressed as mean values ± SEM. Comparisons between two groups were made using unpaired, two-tailed Student’s *t*-test. Analysis of variance (anova) followed by Bonferroni’s multiple comparison test was used for multiple groups. Statistical significance was assigned at a value of *P* < 0.05. All experiments were repeated at least twice.

## Supporting Information

**Fig. S1** Bleomycin exposure activates TGFβ signaling pathway *in vivo*.

**Fig. S2** Age-related changes in oxidized proteins and fibrotic markers after bleomycin exposure.

**Fig. S3** Age-related changes in the cellular levels of autophagosomes after bleomycin injury.

**Fig. S4** Age-related changes in collagen deposition and mitochondria presence inside autophagosomes by region of the lung.

**Fig. S5** TGFβ1 reduces LC3 punctae in lung fibroblasts.

**Fig. S6** TGFβ1 modulates the transcriptional level of autophagy regulator genes during FMD.

**Fig. S7** TGFβ1 reduces autophagic flux of mitochondria.

**Fig. S8** Overexpression of TGFβ1 reduces PINK1 expression *in vivo*.

**Fig. S9** Fibroblasts treated with autophagy inducers resist remodeling effects of TGFβ1.

**Fig. S10** Autophagy inhibition induces expression of fibrotic markers.

**Table S1** Autophagy-related genes deregulated in NHLF by TGFβ1. **Table S2** List of primers used in autophagy array confirmation.

## References

[b1] Aguirre A, Lopez-Alonso I, Gonzalez-Lopez A, Amado-Rodriguez L, Batalla-Solis E, Astudillo A, Blazquez-Prieto J, Fernandez AF, Galvan JA, dos Santos CC, Albaiceta GM (2014). Defective autophagy impairs ATF3 activity and worsens lung injury during endotoxemia. J. Mol. Med. (Berl).

[b2] Akgedik R, Akgedik S, Karamanli H, Uysal S, Bozkurt B, Ozol D, Armutcu F, Yildirim Z (2012). Effect of resveratrol on treatment of bleomycin-induced pulmonary fibrosis in rats. Inflammation.

[b3] Araya J, Kojima J, Takasaka N, Ito S, Fujii S, Hara H, Yanagisawa H, Kobayashi K, Tsurushige C, Kawaishi M, Kamiya N, Hirano J, Odaka M, Morikawa T, Nishimura SL, Kawabata Y, Hano H, Nakayama K, Kuwano K (2013). Insufficient autophagy in idiopathic pulmonary fibrosis. Am. J. Physiol. Lung Cell. Mol. Physiol.

[b4] Biernacka A, Dobaczewski M, Frangogiannis NG (2011). TGF-β signaling in fibrosis. Growth Factors.

[b5] Bitto A, Lerner CA, Nacarelli T, Crowe E, Torres C, Sell C (2014). p62/SQSTM1 at the interface of aging, autophagy, and disease. Age (Dordr).

[b6] Blagosklonny MV (2010). Calorie restriction: decelerating mTOR-driven aging from cells to organisms (including humans). Cell Cycle.

[b7] Bratic A, Larsson NG (2013). The role of mitochondria in aging. J. Clin. Invest.

[b8] Brunk UT, Terman A (2002). The mitochondrial-lysosomal axis theory of aging: accumulation of damaged mitochondria as a result of imperfect autophagocytosis. Eur. J. Biochem.

[b9] Bueno M, Lai YC, Romero Y, Brands J, St Croix CM, Kamga C, Corey C, Herazo-Maya JD, Sembrat J, Lee JS, Duncan SR, Rojas M, Shiva S, Chu CT, Mora AL (2015). PINK1 deficiency impairs mitochondrial homeostasis and promotes lung fibrosis. J Clin Invest.

[b10] Calderilla-Barbosa L, Seibenhener ML, Du Y, Diaz-Meco MT, Moscat J, Yan J, Wooten MW, Wooten MC (2014). Interaction of SQSTM1 with the motor protein dynein–SQSTM1 is required for normal dynein function and trafficking. J. Cell Sci.

[b11] Carrieri G, Marzi E, Olivieri F, Marchegiani F, Cavallone L, Cardelli M, Giovagnetti S, Stecconi R, Molendini C, Trapassi C, De Benedictis G, Kletsas D, Franceschi C (2004). The G/C915 polymorphism of transforming growth factor beta1 is associated with human longevity: a study in Italian centenarians. Aging Cell.

[b12] Chang AL, Ulrich A, Suliman HB, Piantadosi CA (2015). Redox regulation of mitophagy in the lung during murine Staphylococcus aureus sepsis. Free Radic. Biol. Med.

[b13] Chen ZH, Kim HP, Sciurba FC, Lee SJ, Feghali-Bostwick C, Stolz DB, Dhir R, Landreneau RJ, Schuchert MJ, Yousem SA, Nakahira K, Pilewski JM, Lee JS, Zhang Y, Ryter SW, Choi AM (2008). Egr-1 regulates autophagy in cigarette smoke-induced chronic obstructive pulmonary disease. PLoS One.

[b14] Chow JY, Cabral JA, Chang J, Carethers JM (2008). TGFbeta modulates PTEN expression independently of SMAD signaling for growth proliferation in colon cancer cells. Cancer Biol. Ther.

[b15] Deng N, Sanchez CG, Lasky JA, Zhu D (2013). Detecting splicing variants in idiopathic pulmonary fibrosis from non-differentially expressed genes. PLoS One.

[b16] Doyle KP, Cekanaviciute E, Mamer LE, Buckwalter MS (2010). TGFβ signaling in the brain increases with aging and signals to astrocytes and innate immune cells in the weeks after stroke. J. Neuroinflammation.

[b17] Feng Z, Levine AJ (2010). The regulation of energy metabolism and the IGF-1/mTOR pathways by the p53 protein. Trends Cell Biol.

[b18] Geisler S, Holmstrom KM, Skujat D, Fiesel FC, Rothfuss OC, Kahle PJ, Springer W (2010). PINK1/Parkin-mediated mitophagy is dependent on VDAC1 and p62/SQSTM1. Nat. Cell Biol.

[b19] Georgakopoulou EA, Tsimaratou K, Evangelou K, Fernandez MP, Zoumpourlis V, Trougakos IP, Kletsas D, Bartek J, Serrano M, Gorgoulis VG (2013). Specific lipofuscin staining as a novel biomarker to detect replicative and stress-induced senescence. A method applicable in cryo-preserved and archival tissues. Aging (Albany NY).

[b20] Hawkins A, Guttentag SH, Deterding R, Funkhouser WK, Goralski JL, Chatterjee S, Mulugeta S, Beers MF (2015). A non-BRICHOS SFTPC mutant (SP-CI73T) linked to interstitial lung disease promotes a late block in macroautophagy disrupting cellular proteostasis and mitophagy. Am. J. Physiol. Lung Cell. Mol. Physiol.

[b21] He Z, Deng Y, Li W, Chen Y, Xing S, Zhao X, Ding J, Gao Y, Wang X (2014). Overexpression of PTEN suppresses lipopolysaccharide-induced lung fibroblast proliferation, differentiation and collagen secretion through inhibition of the PI3-K-Akt-GSK3beta pathway. Cell Biosci.

[b22] Hecker L, Logsdon NJ, Kurundkar D, Kurundkar A, Bernard K, Hock T, Meldrum E, Sanders YY, Thannickal VJ (2014). Reversal of persistent fibrosis in aging by targeting Nox4-Nrf2 redox imbalance. Sci. Transl. Med.

[b23] Hirose T, Nakano Y, Nagamatsu Y, Misumi T, Ohta H, Ohshima Y (2003). Cyclic GMP-dependent protein kinase EGL-4 controls body size and lifespan in *C. elegans*. Development.

[b24] Honeyman L, Bazett M, Tomko TG, Haston CK (2013). MicroRNA profiling implicates the insulin-like growth factor pathway in bleomycin-induced pulmonary fibrosis in mice. Fibrogenesis Tissue Repair.

[b25] Ichimura Y, Waguri S, Sou YS, Kageyama S, Hasegawa J, Ishimura R, Saito T, Yang Y, Kouno T, Fukutomi T, Hoshii T, Hirao A, Takagi K, Mizushima T, Motohashi H, Lee MS, Yoshimori T, Tanaka K, Yamamoto M, Komatsu M (2013). Phosphorylation of p62 activates the Keap1-Nrf2 pathway during selective autophagy. Mol. Cell.

[b26] Klingsberg RC, Mutsaers SE, Lasky JA (2010). Current clinical trials for the treatment of idiopathic pulmonary fibrosis. Respirology.

[b27] Klionsky DJ, Codogno P, Cuervo AM, Deretic V, Elazar Z, Fueyo-Margareto J, Gewirtz DA, Kroemer G, Levine B, Mizushima N, Rubinsztein DC, Thumm M, Tooze SA (2010). A comprehensive glossary of autophagy-related molecules and processes. Autophagy.

[b28] Knight DA, Ernst M, Anderson GP, Moodley YP, Mutsaers SE (2003). The role of gp130/IL-6 cytokines in the development of pulmonary fibrosis: critical determinants of disease susceptibility and progression?. Pharmacol. Ther.

[b29] Korfei M, Schmitt S, Ruppert C, Henneke I, Markart P, Loeh B, Mahavadi P, Wygrecka M, Klepetko W, Fink L, Bonniaud P, Preissner KT, Lochnit G, Schaefer L, Seeger W, Guenther A (2011). Comparative proteomic analysis of lung tissue from patients with idiopathic pulmonary fibrosis (IPF) and lung transplant donor lungs. J. Proteome Res.

[b31] Nho RS, Hergert P (2014). IPF fibroblasts are desensitized to type I collagen matrix-induced cell death by suppressing low autophagy via aberrant Akt/mTOR kinases. PLoS One.

[b32] Pardo A, Selman M (2002). Idiopathic pulmonary fibrosis: new insights in its pathogenesis. Int. J. Biochem. Cell Biol.

[b33] Patel AS, Lin L, Geyer A, Haspel JA, An CH, Cao J, Rosas IO, Morse D (2012). Autophagy in idiopathic pulmonary fibrosis. PLoS One.

[b34] Peng R, Sridhar S, Tyagi G, Phillips JE, Garrido R, Harris P, Burns L, Renteria L, Woods J, Chen L, Allard J, Ravindran P, Bitter H, Liang Z, Hogaboam CM, Kitson C, Budd DC, Fine JS, Bauer CM, Stevenson CS (2013). Bleomycin induces molecular changes directly relevant to idiopathic pulmonary fibrosis: a model for “active” disease. PLoS One.

[b35] Pyo JO, Yoo SM, Ahn HH, Nah J, Hong SH, Kam TI, Jung S, Jung YK (2013). Overexpression of Atg5 in mice activates autophagy and extends lifespan. Nat. Commun.

[b36] Roe ND, Xu X, Kandadi MR, Hu N, Pang J, Weiser-Evans MC, Ren J (2015). Targeted deletion of PTEN in cardiomyocytes renders cardiac contractile dysfunction through interruption of Pink1-AMPK signaling and autophagy. Biochim. Biophys. Acta.

[b37] Schiavi A, Ventura N (2014). The interplay between mitochondria and autophagy and its role in the aging process. Exp. Gerontol.

[b38] Sener G, Topaloglu N, Sehirli AO, Ercan F, Gedik N (2007). Resveratrol alleviates bleomycin-induced lung injury in rats. Pulm. Pharmacol. Ther.

[b39] Shoji-Kawata S, Sumpter R, Leveno M, Campbell GR, Zou Z, Kinch L, Wilkins AD, Sun Q, Pallauf K, MacDuff D, Huerta C, Virgin HW, Helms JB, Eerland R, Tooze SA, Xavier R, Lenschow DJ, Yamamoto A, King D, Lichtarge O, Grishin NV, Spector SA, Kaloyanova DV, Levine B (2013). Identification of a candidate therapeutic autophagy-inducing peptide. Nature.

[b40] Sueblinvong V, Neujahr DC, Mills ST, Roser-Page S, Ritzenthaler JD, Guidot D, Rojas M, Roman J (2012). Predisposition for disrepair in the aged lung. Am. J. Med. Sci.

[b41] Sueblinvong V, Neveu WA, Neujahr DC, Mills ST, Rojas M, Roman J, Guidot DM (2014). Aging promotes pro-fibrotic matrix production and increases fibrocyte recruitment during acute lung injury. Adv. Biosci. Biotechnol.

[b42] Taguchi T, Razzaque MS (2007). The collagen-specific molecular chaperone HSP47: is there a role in fibrosis?. Trends Mol. Med.

[b43] van der Kraan PM, Goumans MJ, Blaney Davidson E, ten Dijke P (2012). Age-dependent alteration of TGF-β signalling in osteoarthritis. Cell Tissue Res.

[b44] Vestergaard PF, Hansen M, Frystyk J, Espelund U, Christiansen JS, Jorgensen JO, Fisker S (2014). Serum levels of bioactive IGF1 and physiological markers of ageing in healthy adults. Eur. J. Endocrinol.

[b45] Zhao J, Shi W, Wang YL, Chen H, Bringas P, Datto MB, Frederick JP, Wang XF, Warburton D (2002). Smad3 deficiency attenuates bleomycin-induced pulmonary fibrosis in mice. Am. J. Physiol. Lung Cell. Mol. Physiol.

