# Supplementary figures and images for: Deregulation of selective autophagy during aging and pulmonary fibrosis: the role of TGFβ1
Source: Aging Cell. 2015 Jun 9;14(5):774–83. doi: 10.1111/acel.12357 (PMC4568965; doi:10.1111/acel.12357)

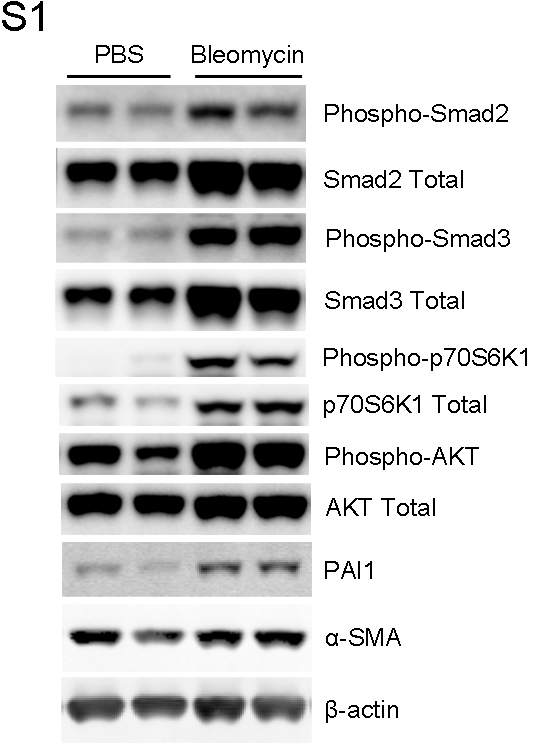

Supplement: Supplementary file 1 [file acel0014-0774-sd1.tif]

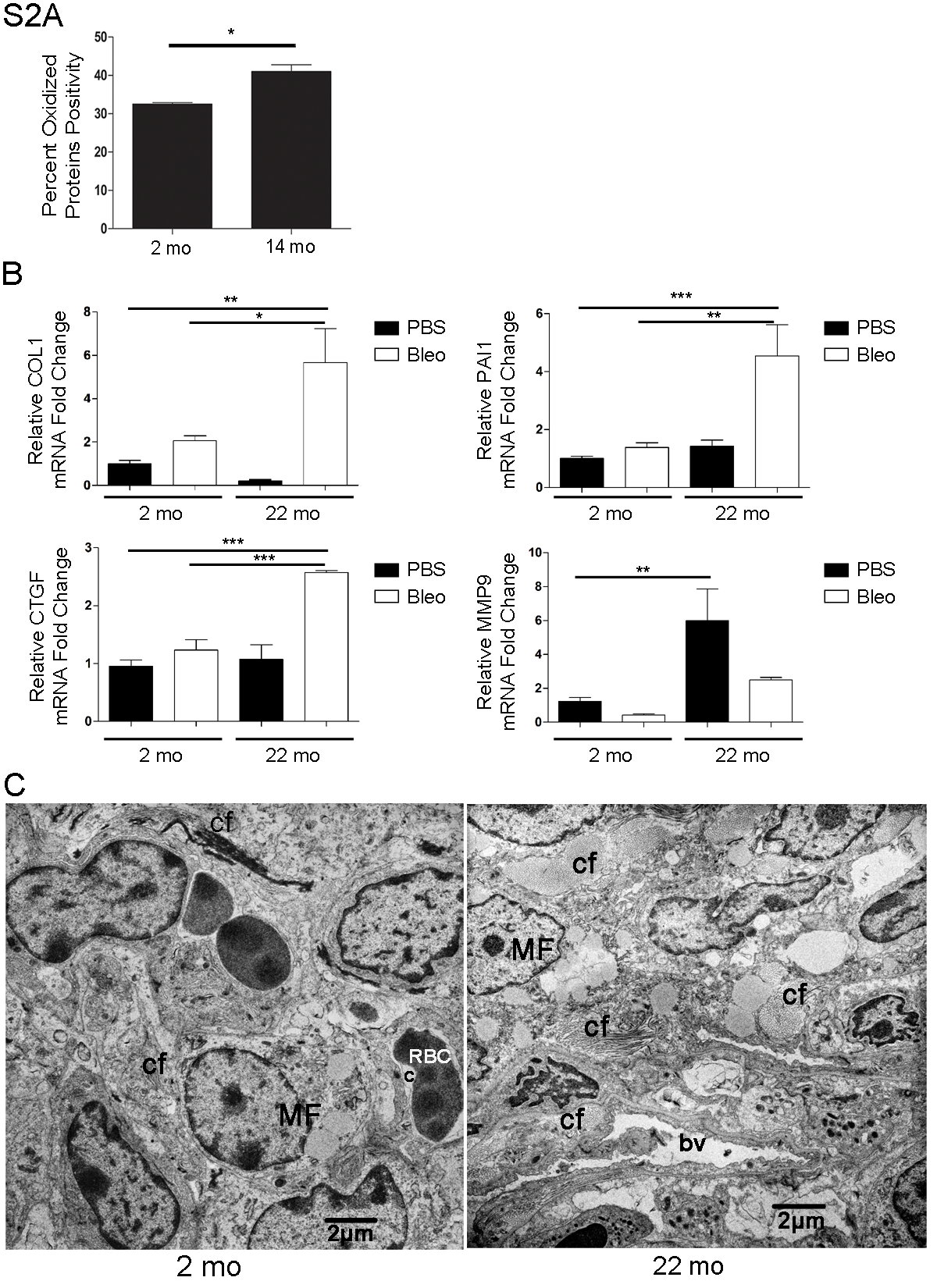

Supplement: Supplementary file 2 [file acel0014-0774-sd2.tif]

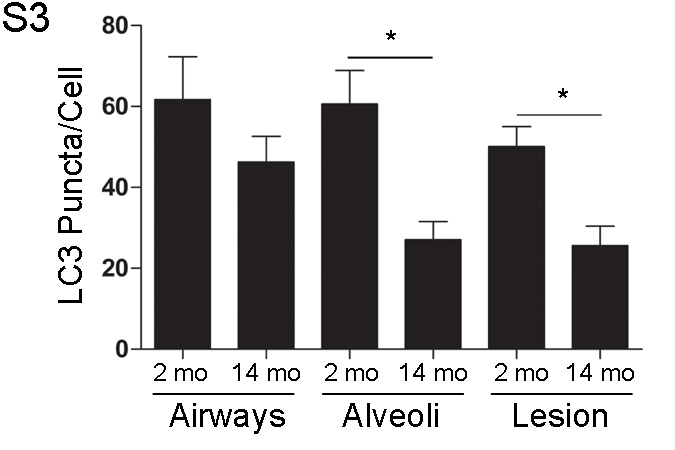

Supplement: Supplementary file 3 [file acel0014-0774-sd3.tif]

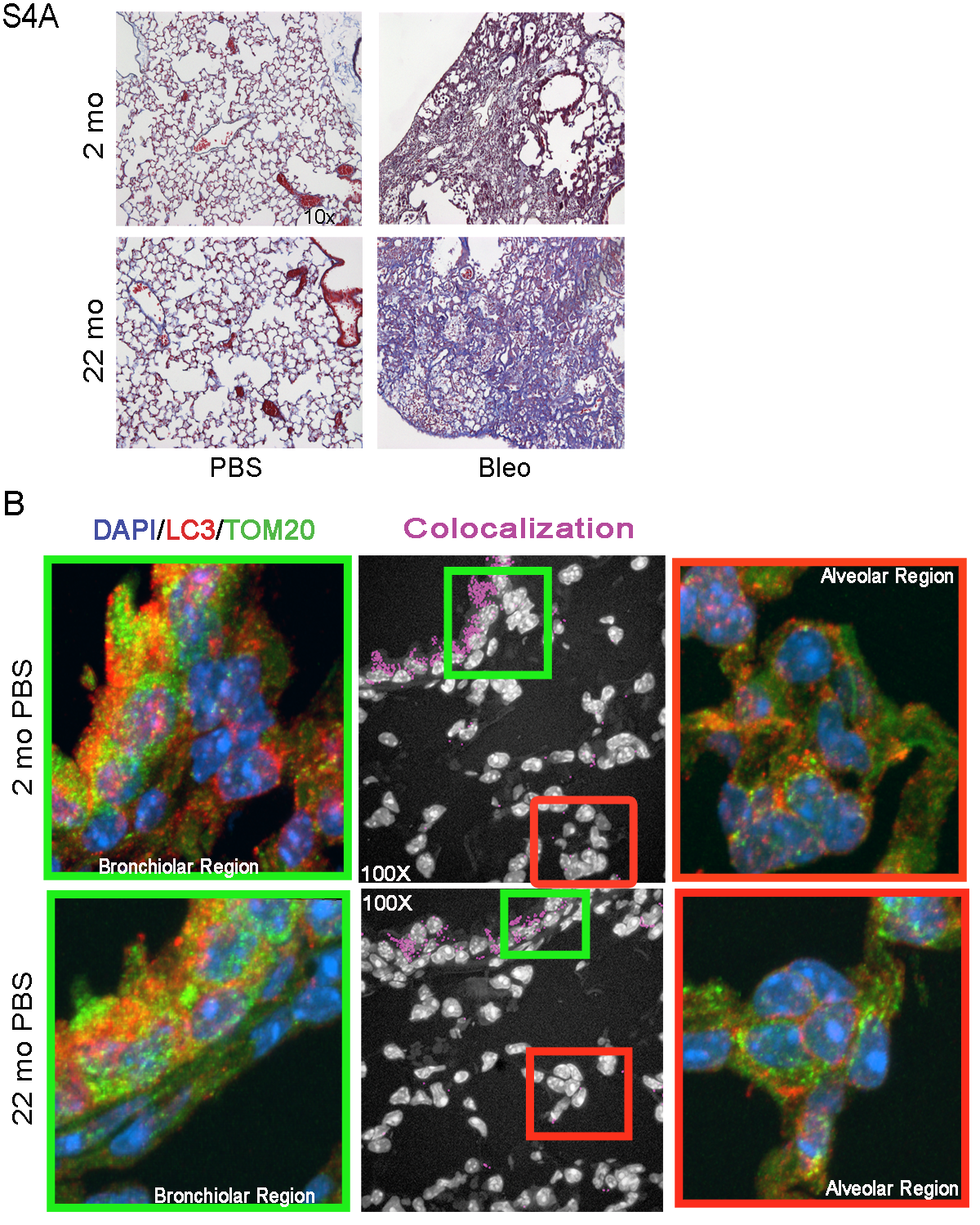

Supplement: Supplementary file 4 [file acel0014-0774-sd4.tif]

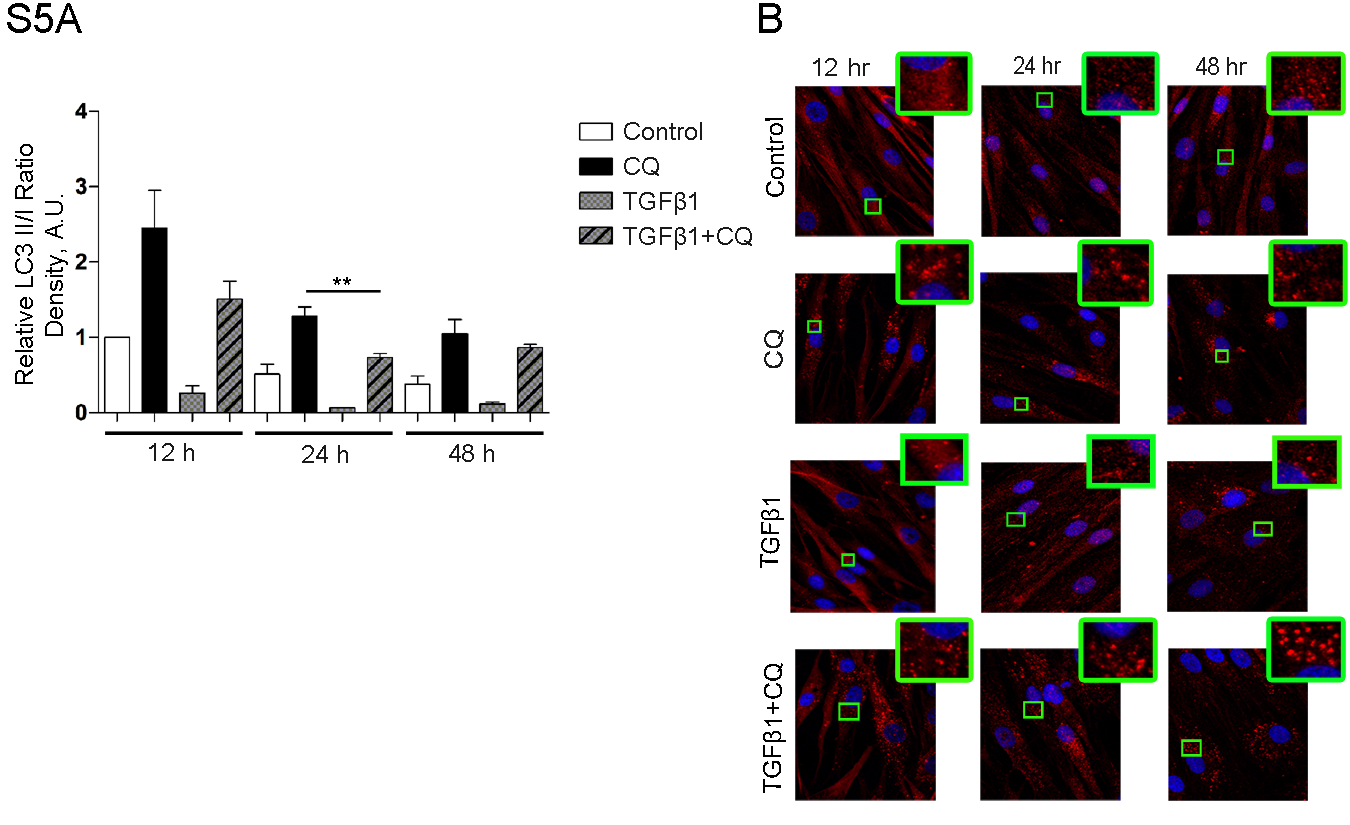

Supplement: Supplementary file 5 [file acel0014-0774-sd5.tif]

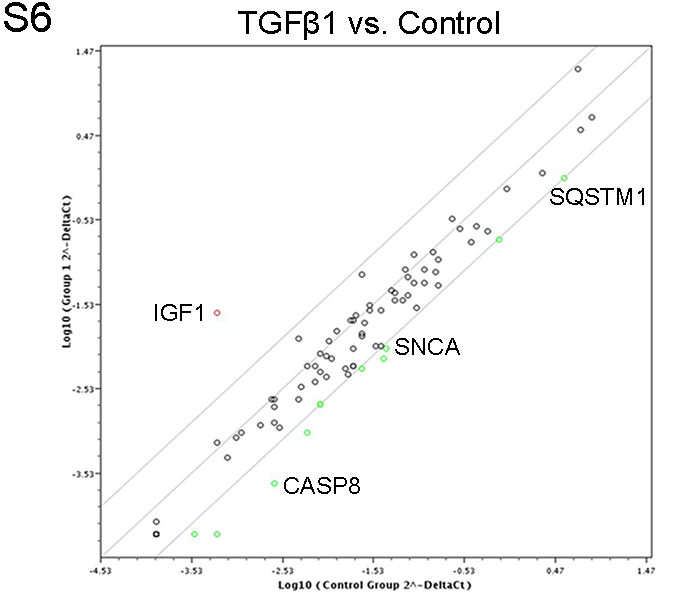

Supplement: Supplementary file 6 [file acel0014-0774-sd6.tif]

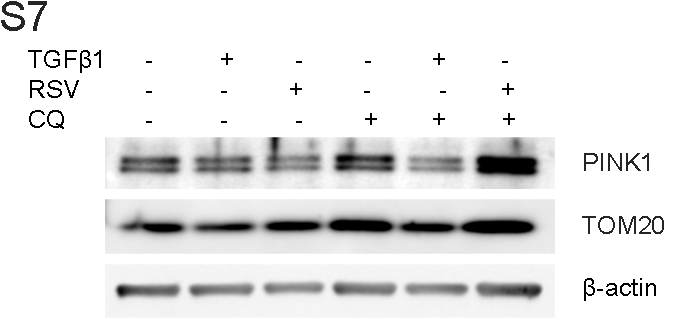

Supplement: Supplementary file 7 [file acel0014-0774-sd7.tif]

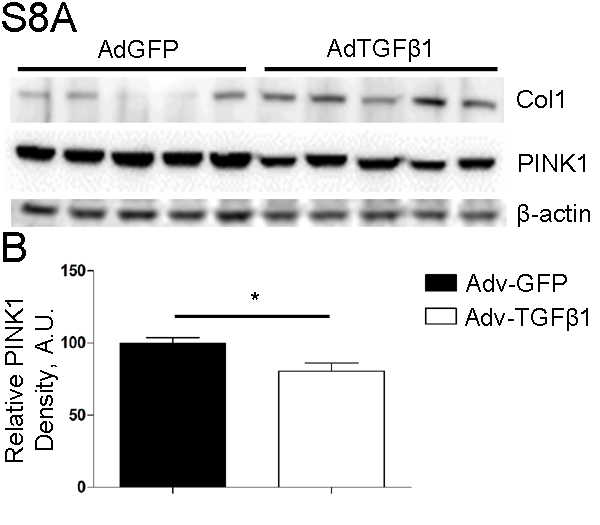

Supplement: Supplementary file 8 [file acel0014-0774-sd8.tif]

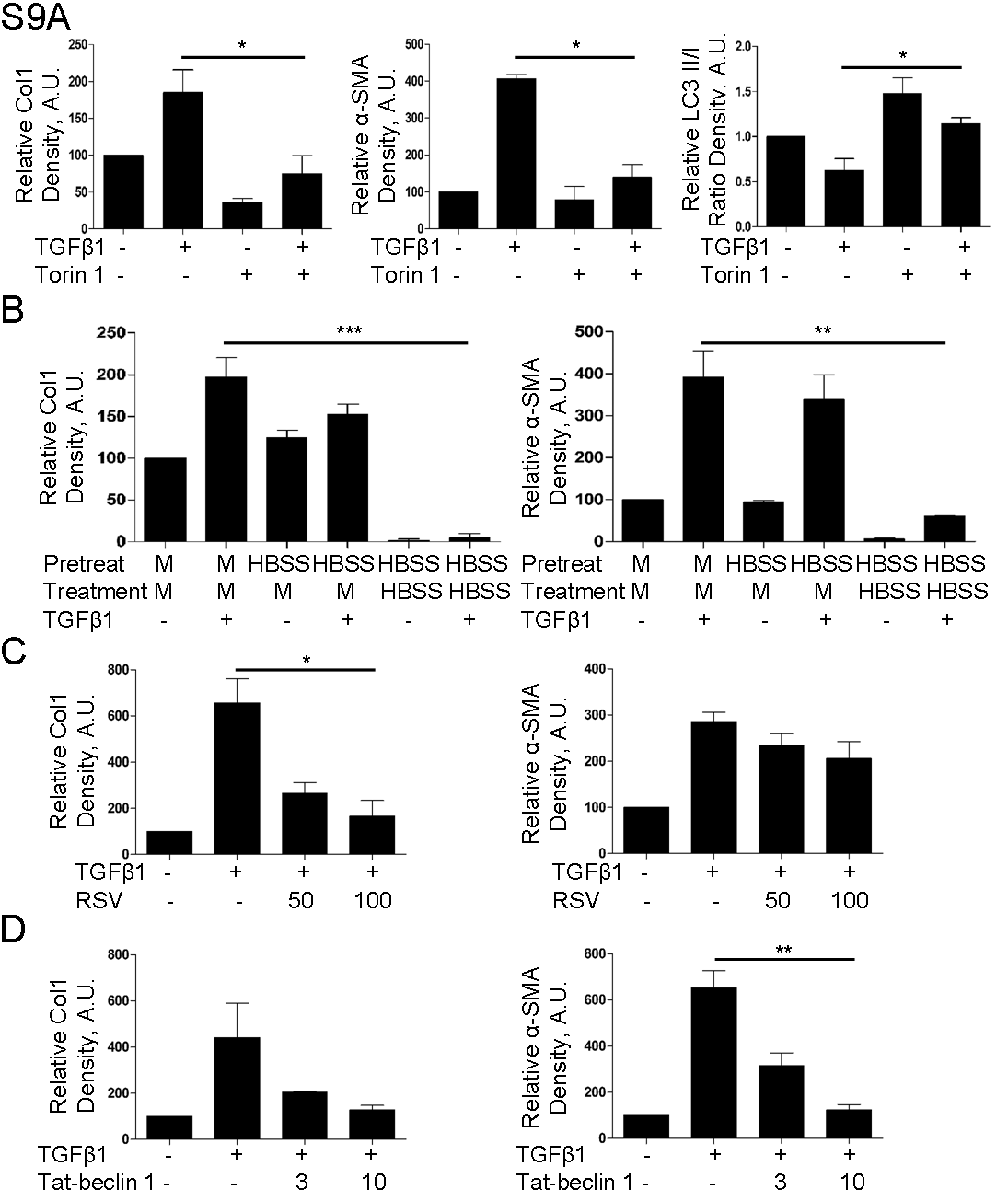

Supplement: Supplementary file 9 [file acel0014-0774-sd9.tif]

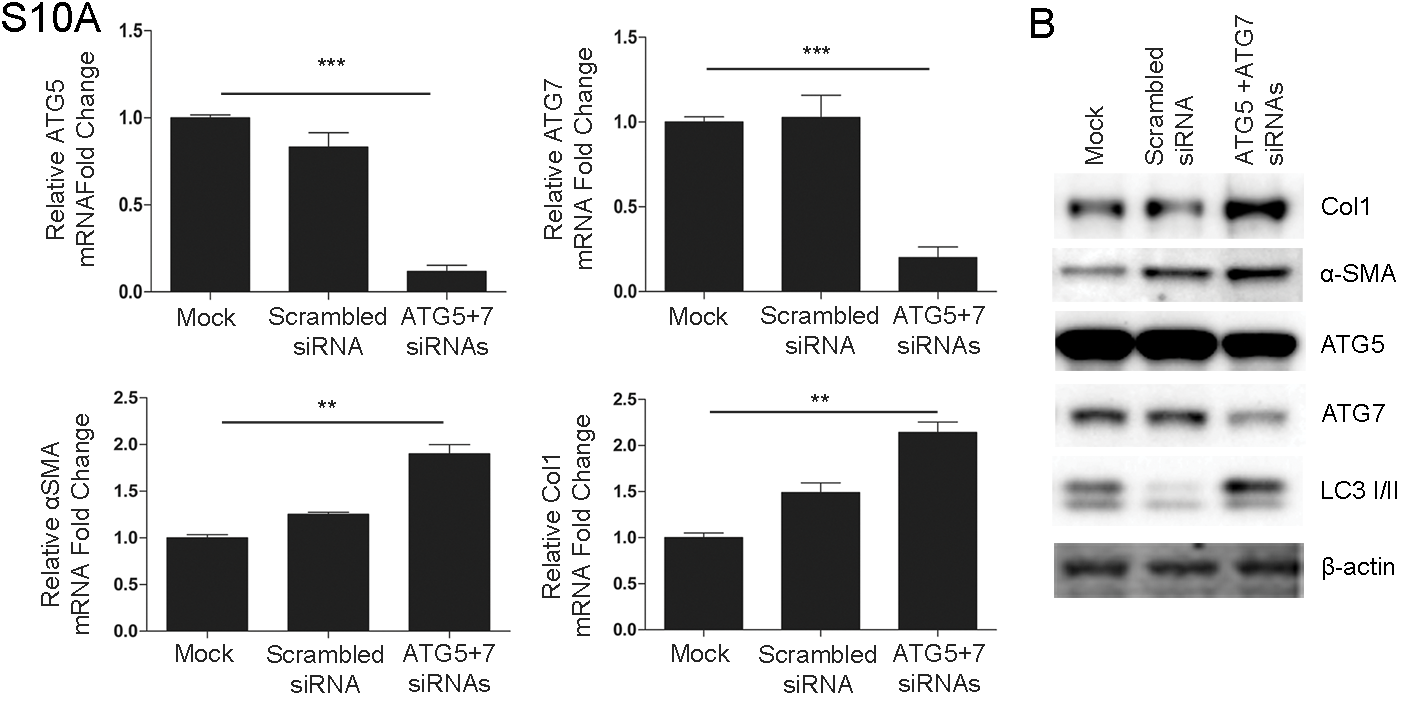

Supplement: Supplementary file 10 [file acel0014-0774-sd10.tif]
