# Supplementary material for: Deregulation of selective autophagy during aging and pulmonary fibrosis: the role of TGFβ1
Source: Aging Cell. 2015 Jun 9;14(5):774–83. doi: 10.1111/acel.12357 (PMC4568965; doi:10.1111/acel.12357)
Supplement: Supplementary file 11 [file acel0014-0774-sd11.docx]

Supplemental Experimental Procedures.

Supporting Information Figures listed below.

**Fig. S1.** Bleomycin exposure activates TGFβ signaling pathway in vivo.

**Fig. S2.** Age-related changes in oxidized proteins and fibrotic markers after bleomycin exposure.

**Fig. S3.** Age-related changes in the cellular levels of autophagosomes after bleomycin injury.

**Fig. S4.** Age-related changes in collagen deposition and mitochondria presence inside autophagosomes by region of the lung.

**Fig. S5.** TGFβ1 reduces LC3 punctae in lung fibroblasts.

**Fig. S6.** TGFβ1 modulates the transcriptional level of autophagy regulator genes during FMD.

**Fig. S7.** TGFβ1 reduces autophagic flux of mitochondria.

**Fig. S8.** Overexpression of TGFβ1 reduces PINK1 expression in vivo.

**Fig. S9.** Fibroblasts treated with autophagy inducers resist remodeling effects of TGFβ1.

**Fig. S10.** Autophagy inhibition induces expression of fibrotic markers.

**Table S1.** Autophagy-related genes deregulated in NHLF by TGFβ1.

**Tables**

| Gene symbol | Fold regulation  1 ng/ml TGFβ1 treatment versus DMSO control |
| --- | --- |
| AKT1 | -1.5369 |
| AMBRA1 | -2.1735 |
| APP | -2.1735 |
| ARSA | -2.0279 |
| ATG10 | 1.2142 |
| ATG12 | -1.0867 |
| ATG16L1 | -1.8921 |
| ATG16L2 | -2.6759 |
| ATG3 | 1.1329 |
| ATG4A | -1.2483 |
| ATG4B | -2.0279 |
| ATG4C | -1.6472 |
| ATG4D | -1.1647 |
| ATG5 | -1.08867 |
| ATG7 | -3.2944 |
| ATG9A | -1.0867 |
| ATG9B | -2.1735 |
| BAD | -4.3469 |
| BAK1 | -1.014 |
| BAX | -2.0279 |
| BCL2 | -10.7034 |
| BCL2L1 | -6.1475 |
| BECN1 | -1.434 |
| BID | -3.2944 |
| BNIP3 | 1.3947 |
| CASP3 | 1.057 |
| CASP8 | -1.1647 |
| CDKN1B | -1.7654 |
| CDKN2A | -1.8921 |
| CLN3 | -2.8679 |
| CTSB | -4.0558 |
| CTSS | -3.7842 |
| CXCR4 | -2.1735 |
| DAPK1 | -9.9866 |
| DRAM1 | -3.2944 |
| EIF2AK3 | 1.1329 |
| EIF4G1 | -1.3379 |
| ESR1 | -2.1735 |
| FADD | -1.014 |
| FAS | -1.5369 |
| GAA | -3.5308 |
| GABARAP | -2.0279 |
| GABARAPL1 | -1.434 |
| GABARAPL2 | 1.3947 |
| HDAC1 | -2.0279 |
| HGS | -1.6472 |
| HSP90AA1 | -2.4967 |
| HSPA8 | -1.2483 |
| HTT | -4.0558 |
| IFNA2 | -1.5369 |
| IFNA4 | -2.1735 |
| IFNG | -2.1735 |
| IGF1 | 41.6429 |
| INS | -2.1735 |
| IRGM | -2.1735 |
| MAP1LC3A | -3.2944 |
| MAP1LC3B | -1.0867 |
| MAPK14 | -1.6472 |
| MAPK8 | 1.057 |
| NFKB1 | -1.5369 |
| PIK3C3 | -1.2483 |
| PIK3CG | -2.1735 |
| PIK3R4 | -2.0279 |
| PRKAA1 | 1.1329 |
| PRKAA2 | -1.5369 |
| PTEN | -1.1647 |
| RAB24 | -1.5369 |
| RB1 | 1.1329 |
| RGS19 | -1.3379 |
| RPS6KB1 | -1.2483 |
| SNCA | -4.5489 |
| SQSTM1 | -4.0558 |
| TGFB1 | 2.9897 |
| TGM2 | -1.1647 |
| FAM176A | 2.6027 |
| TMEM74 | -2.1735 |
| DRAM2 | -3.0738 |
| TNF | -5.7358 |
| TNFSF10 | -5.7358 |
| TP53 | -1.8921 |
| TP73 | -2.1735 |
| ULK1 | -1.014 |
| ULK2 | -4.0558 |
| UVRAG | -1.5369 |

*Table S1. Autophagy-related genes deregulated in NHLF by TGFB1. The list of genes’ fold changes between untreated and treated with 1 ng/ml TGFβ1.

**Supporting Information Figure Legends**

Figure S1. **Bleomycin exposure activates TGFβ signaling pathway in vivo.** Representative Western blots (WB) from young (2 month old) mice exposed to vehicle PBS control or bleomycin for TGFβ signaling pathway activation markers phosphorylated Smad2/3 and AKT, as well as fibrotic markers, plasminogen activator inhibitor-1 (PAI1), and α-smooth muscle actin (α-SMA). β-actin was used as a loading control.

Figure S2. **Age-related changes in expression of fibrotic markers after bleomycin injury.** A) Quantification of accumulated oxidized proteins in young (2 month old) and middle-aged (14 month old) lung after oropharyngeal aspiration of bleomycin (Bleo) or PBS vehicle only, at 14 days postexposure. B) qRT–PCR analysis for fibrotic markers COL1, PAI1, CTGF, and MMP9 expression in 2-month-old (n=5) and 22-month-old mice (n=4) after Bleo exposure. C) Representative electron microscopy images in 2-month-old and 22-month-old mice post-Bleo exposure showing collagen fibrils. Collagen fibrils labeled as cf, blood vessels as bv, myofibroblasts as MF, red blood cells as RBC, and capillaries as c. *P<0.05, **P<0.01, ***P<0.005.

Fig. S3. **Age-related changes in the cellular levels of autophagosomes after bleomycin injury**. LC3 punctae quantification in 2-month-old and 14-month-old mice in different sections of the lung tissue, including airways, alveolar epithelium, and fibrotic tissue. *P<0.05.

Figure S4. **Age-related changes in collagen deposition and mitochondria presence inside autophagosomes by region of the lung.** A) Representative images and quantification of Masson’s trichrome staining to evaluate collagen deposition in young (2 month old) and old (22 month old) after oropharyngeal aspiration of bleomycin (Bleo) or PBS vehicle only, at 14 days postexposure. Positive collagen deposition appears blue. B) Representative images of the basal level of colocalization of LC3 punctae and TOM20, a mitochondria marker, in young (2 month old) and old (22 month old) PBS control mouse lung tissue. Yellow points indicate mitochondria inside autophagosomes in merged color images as positive LC3 staining appears as red punctae and TOM20 green. Nuclei counterstained with DAPI appear blue. Colocalization points appear pink in black and white image. Highlighted green square focuses on bronchiolar region of lung. Highlighted red square focuses on alveolar region of lung.

Fig. S5. **TGFβ1 reduces LC3 punctae in lung fibroblasts.** A) Densitometry analysis of Western blot (WB) from NHLF cultured for 12, 24, and 48 h with and without TGFβ1 and/or chloroquine (CQ) for LC3 II/LC3 I ratio expression shown in Figure 3C. Arbitrary units abbreviated as AU. B) Representative immunofluorescence images for LC3 punctae in NHLF treated for 12, 24, and 48 h with TGFβ1 and 4 h with chloroquine (CQ) to investigate the autophagy flux. **P<0.01.

Fig. S6. **TGFβ1 modulates the transcriptional level of autophagy regulator genes during FMD.** Gene expression array for autophagy-related genes comparing NHLF treated with or without TGFβ1 for 24 h.

Fig. S7. **TGFβ1 reduces autophagic flux of mitochondria.** NHLF control or treatment with TGFβ1 and/or resveratrol (RSV) for 24 h. Chloroquine (CQ) added 4 h prior to collection for autophagic flux analysis. Representative Western blots (WB) for PINK1 and TOM20 expression. β-actin was used as a loading control.

Fig. S8. **Overexpression of TGFβ1 reduces PINK1 expression in vivo.** Oropharyngeal aspiration of adenovirus-TGFβ1 modulates PINK1 expression in vivo. A) Representative WB for collagen type I (Col1) and PINK1 expression from adenovirus-TGFβ1 (AdTGFβ1) or adenovirus-GFP (AdGFP) control infection in mouse lung, at 7 days postinfection. β-actin used as loading control. B) Densitometry analysis of PINK1 expression is shown in Figure 5F. Arbitrary Units abbreviated as AU. *P<0.05.

Fig. S9. **Fibroblasts treated with autophagy inducers resist the remodeling effects of TGFβ1.** A) Densitometry analysis of Western blot (WB) from NHLF treated with TGFβ1 and/or Torin 1 for fibrotic markers, collagen type I (Col1) and α-SMA expression shown in Figure 6A. B) Densitometry analysis of WB from NHLF treated with TGFβ1 and/or starvation conditions for fibrotic markers shown in Figure 6B. C) Densitometry analysis of WB from NHLF treated with TGFβ1 and/or resveratrol (RSV) for fibrotic markers shown in Figure 6C. D) Densitometry analysis of WB from NHLF treated with TGFβ1 and/or Tat-beclin 1 peptide for fibrotic markers is shown in Figure 6D. Arbitrary Units abbreviated as AU. *P<0.05, **P<0.01, ***P<0.005.

Figure S10. **Autophagy inhibition induces expression of fibrotic markers.** A) qRT–PCR analysis from NHLF transfected with siRNA for ATG5 and ATG7 to evaluate transcriptional changes in ATG5, ATG7, and fibrotic markers collagen type I (Col1) and α-SMA expression. B) Representative Western blots for Col1, α-SMA, ATG5, ATG7, and LC3 in NHLF deficient in ATG5 and ATG7. **P<0.01, ***P<0.005.

**Supplemental Experimental Procedures**

**Cell Culture, Reagents, & Transfection**

For cell culture experiments, recombinant human TGFβ1 (R&D Systems, Minneapolis, MN, USA) was used at a concentration of 1 ng/mL. For calorie restriction, Hank's Balanced Salt Solution (HBSS, Life Technologies, Grand Island, NY, USA) was used. Torin 1 obtained from Selleck Chemicals (Houston, TX, USA) was used at a concentration of 250 nM. Tat-beclin 1 peptide was obtained from Phoenix Pharmaceuticals, Inc. (Burlingame, CA, USA) and used at concentrations of 3 and 10 µM. Resveratrol from Sigma (St. Louis, MO, USA) was used at concentrations of 50 and 100 μM. Chloroquine (Sigma) was used at 30 μM for at least 4 h. For siRNA knockdown experiments, 1x10^6^ cells per transfection were harvested and washed once in PBS (Invitrogen) then resuspended in 100 μl of resuspension buffer R with 5 μM siGENOME SMARTpool ATG5 (M-004374-04) & ATG7 (M-020112-01) targeted siRNA (Dharmacon RNAi Technologies Inc., Lafayette, CO, USA), 5 μM control nonsilencing siRNA (D-001210-01) or sterile water for mock transfection. This mix was transfected in 100 μl using two pulse of 1400 V input pulse voltage and 20 ms input pulse width. Transfected NHLFs were plated on 60-mm tissue culture dishes in 5 ml of FGM-2 medium for 48 h.

**Mice & Tissue Samples**

Animals were anesthetized with 2% isoflurane vapor (VetOne, Meridian, ID, USA) in oxygen. Treatments were administered in 50 μl of sterile PBS by oropharyngeal aspiration. C57BL/6 mice aged young (2 month old, n=5 per treatment), middle aged (14 month old, n=5), and old (22 month old, n=5 per treatment) received 2 U/kg Bleomycin (Teva Parenteral Medicines, Irvine, CA, USA) or vehicle only (PBS) for control. C57BL/6 mice aged 6–8 weeks were treated with 3 x 10^8^PFU of replication-deficient adenovirus encoding either GFP (control group, AdGFP, n=5) or active TGF-β1 (AdTGF-β1, n=5). Animals were anesthetized with 80 mg/kg ketamine plus 8 mg/kg xylazine and euthanized by exsanguination 14 or 7 days after bleomycin or adenovirus treatments, respectively. The right lungs were snap-frozen in liquid nitrogen and stored at -80°C for RNA or protein isolation. The left lungs were inflation-fixed with 10% neutral buffered formalin (Sigma-Aldrich, Sigma-Aldrich Corp., St. Louis, MO, USA) through the trachea at 25 cm H_2_O pressure for 15 min, excised from the mice, and stored in fresh 10% neutral buffered formalin at 4°C for histology. At least two independent experiments were performed and analyzed. Sections from human IPF and control lung specimens were obtained from the NIH Lung Tissue Research Consortium (LTRC).

**Western Blots**

Protein samples combined with 4x NuPAGE LDS Sample Buffer and 10x NuPAGE Sample Reducing Agent (Invitrogen) for a final concentration of 1x for both buffers and boiled for 5 minutes. 20–25 micrograms of each protein sample was separated on NuPage SDS 4-12% Bis-Tris gradient gels (Invitrogen) and transferred onto PVDF membranes (Invitrogen). Mitochondria samples were combined for a final concentration of 1x LDS sample buffer and separated on aforementioned gels without boiling. Rat heart mitochondria provided with OXPHOS antibody (information listed below, MitoSciences, Abcam, Cambridge, MA, USA) was used as a positive control. For time-dependent cell lysates, protein samples were separated on 15% acrylamide 1.5M Tris/10% SDS gel. Membranes were blocked in 5% BSA in TBST or 5% nonfat dry milk (Blotting-Grade Blocker, Bio-Rad Laboratories Inc., Hercules, CA, USA) in TBST for 1 h at RT then probed with primary antibody overnight at 4°C while shaking. Western blots were imaged using ImageQuant LAS 4000 (GE Healthcare, Pittsburgh, PA, USA) or LiCor imaging systems.  Densitometry analysis was performed using National Institutes of Health (NIH) ImageJ 1.48d (Wayne Rasband NIH, USA, http:imagej.nih.gov/ij) software.

**Antibodies**

Antibodies to Collagen-1, PINK1, BNIP3L and p62/SQSTM1 were purchased from Abcam (Western blot, 1:1000; Cambridge, MA, USA). Antibodies to ATG7, Phospho-AKT (S473), AKT, Phospho-p70 S6 Kinase (T389), p70 S6 Kinase, Phospho-Smad2 (S465/467), Smad2, Phospho-Smad3 (S423/425), Smad3, and β-actin were purchased from Cell Signaling (1:1000; Danvers, MA, USA). Antibodies to TOM20 and Hsp47 (H300) were purchased from Santa Cruz Biotechnology (1:500; Dallas, TX, USA). Antibody to Pai1 was purchased from PeproTech (1:10,000, Rocky Hill, NJ, USA). Antibody to α-smooth muscle actin (SMA) was purchased from Sigma (1:10,000). Antibodies to ATG5 and LC3 were purchased from MBL International (1:500; Woburn, MA, USA). MitoProfile total OXPHOS rodent WB antibody cocktail (MitoSciences) was used for immunodetection of the 5 OXPHOS complexes at a dilution of 1:250. Anti-mouse IgG, HRP-linked, anti-rabbit IgG, HRP-linked (1:15,000; Cell Signaling) for chemiluminescent detection and IRDye 800CW goat anti-rabbit IgG or IRDye 680 goat anti-mouse IgG (1:15,000; LiCor, Lincoln, NE, USA) for fluorescent detection.

**Electron Microscopy**

NHLFs cultured on 10 cm^2^ plates were fixed in 2.5% glutaraldehyde in 0.1 M sodium cacodylate buffer pH 7.4 for 1 h at room temperature. Samples were washed three times for 5 m in 0.1 M sodium cacodylate buffer and sent to the Electron Microscopy Core Facility of the Department of Cell Biology at Yale University School of Medicine (New Haven, CT). The Yale core facility provides images and grids for subsequent analysis with the Tecnai G2 F30 TWIN 300 kV/FEG Transmission Electron Microscope (FEI, Hillsboro, OR, USA) at Tulane University.

**Histology, Immunofluorescence–Paraffin Staining, and Immunohistochemistry**

Masson’s trichrome staining was performed as previously described in [Sueblinvong *et al.* 2012](#_ENREF_48). For immunohistochemical samples, slides were incubated in BLOXALL™ Blocking solution (Vector Laboratories, Burlingame, CA, USA) for 10 m before proceeding with antigen retrieval. Slides were then incubated for 20 m in 0.5% ammonium chloride and rinsed in PBS then incubated in 0.3M glycine for 10 m. Slides were blocked in HEPES NaCl buffer (20 mM HEPES, 1% BSA, 135 mM NaCl) for 5 min then incubated for 30 m with blocking serum. The primary antibodies of Hsp47 (H300) and PINK1 were used at dilutions of 1:50 and 1:250, respectively, and were incubated for 30 m followed by diluted biotinylated secondary antibody for 30 m. Slides were then incubated with NovaRed Vectastain®ABC Reagent (Vector Laboratories) for 30 m; then, nuclei were counterstained with hematoxylin and mounted. For immunofluorescence samples, slides were permeabilized in 0.2% Trition-X in TBS buffer for 45 m at RT and were washed in TBS after blocking with 10% BSA in TBS (blocking serum) for 1 h; the primary antibody was added. Antibodies to LC3 (rabbit) and TOM20 (mouse) were used at a dilution of 1:200 and incubated overnight at 4°C in a humidified chamber. Secondary antibodies AlexaFluor 594 goat anti-rabbit IgG (Invitrogen) and AlexaFluor 488 goat anti-mouse IgG (Invitrogen) were employed for immunofluorescence detection at a dilution of 1:1000. Nuclei were counterstained using DAPI (Invitrogen) before mounting in Prolong Gold antifade media (Invitrogen).

**Oxidative Stress Assays**

In tissues, the OxyIHC™ Oxidative Stress Detection Kit (Millipore, Billerica, MA, USA) was used to observe protein oxidation in lung tissue. Slides were immersed in heated sodium citrate buffer for 15 m then transferred to 0.3M glycine for 30 m and methacarn fixed overnight before proceeding according to manufacturer's instructions.

**Imaging & Quantitative Analysis**

For electron microscopy analysis, the number of autophagic vacuoles per cell body was counted using EM images at direct magnification of 4500x and 12,000x for the various treatment conditions. Electron micrographs (control n=10, TGFβ1=12, TGFβ1+RSV=6) were examined, and values are expressed as AVs per field. All numerical values are expressed as mean ± SEM. For histology and immunohistochemistry analysis, images were captured with a Scan Scope Aperio, version 10.2.0.0 Aperio Technologies (Leica Biosystems, Buffalo Grove, IL, USA) at the same magnification with similar contrast. TIFF file images were then exported and analyzed using ImageJ ([imagej.nih.gov](http://imagej.nih.gov/ij/download/)). For quantification of Sudan Black B staining, images were inverted, and applied threshold and positive areas were measured. For quantification of Hsp47 staining, color images were deconvoluted, and positive red color areas measured with the result expressed as the fraction of positive-stained pixels over total area were analyzed. For immunofluorescence, images were captured in Z-stacks using Nikon A1+ Inverted Confocal microscope (Nikon Instruments Inc., Melville, NY, USA). Three-dimensional reconstruction and volume rendering of Z-stacks were carried out with appropriate ImageJ plug-in and NIS element AR 4.30 (Nikon) software. For quantification of LC3 punctae, images were captured with an Olympus BX60 microscope equipped with epifluorescence optics (Olympus, Melville, NY) and coupled device camera Magnafire (MagnaFire 2.6; Olympus) with a barrier filter equipped for simultaneous detection of FITC, Texas red and DAPI. TIFF files were normalized, merged and analyzed with Image J. The number of LC3 dots was counted in at least five independent visual fields at 100x oil objective of magnification. The results were expressed as puncta per cell. For quantification of mitochondria within autophagosomes, the colocalization of autophagosomes (LC3 punctae) and mitochondria (TOM20) was detected using the colocalization finder plug-in in Image J.

**RNA Isolation, qRT–PCR & Gene Expression Array**

RNA concentration was measured using NanoDrop spectrophotometer (Thermo Scientific Nanodrop, Nanodrop Technologies, Wilmington, DE, USA). For quantitative real-time PCR analysis, 1 μg of total RNA was reverse transcribed using iScript^TM^ cDNA Synthesis Kit (Bio-Rad), according to manufacturer’s protocol. Quantitative real-time PCRs were performed using iQ^TM^SYBR® Green Supermix (Bio-Rad) in the Bio-Rad MyiQ iCycler (Hercules, CA). Relative expression levels were calculated using the 2^-ΔΔC(T)^ method and normalized to 36b4 expression. For gene expression array studies, 1 μg of total RNA was reversed transcribed using the RT^2^ First Strand Kit (SABiosciences) and resulting cDNA was added to RT^2^qPCR Mastermix (SABiosciences) at correct dilutions and aliquoted to PCR array. The real-time PCR was performed using MyiQ iCycler (Bio-Rad). Analysis of the PCR array profile was performed using web-based software RT^2^ Profiler PCR Array Data Analysis version 3.5 provided at the SABiosciences website (<http://pcrdataanalysis.sabiosciences.com/pcr/arrayanalysis.php>). Genes that demonstrated a fold change >3 were selected for further study with primer information given in table below.

| Gene | Sequence or catalog number from realtimeprimers.com |
| --- | --- |
| ATG5 | Forward 5’-ATGCAGGGAACACTAAGCTG-3’  Reverse 5’-TCTAGGGCATTGTAGGCTTG-3’ |
| ATG16L1 | HATPL-I |
| ATG16L2 | HATPL-I |
| ATG4C | HATPL-I |
| ATG7 | Forward 5’-TTGAGCGGCGGCAAGAAATAATGG-3’  Reverse 5’-AGCTTCATCCAGCCGATACTCGTT-3’ |
| BAD | Forward 5’-TGACGAGTTTGTGGACTCCT-3’  Reverse 5’-TACTTCCGCCCATATTCAAG-3’ |
| BCL2 | Forward 5’-ATTTCCTGCATCTCATGCCAAGGG-3’  Reverse 5’-TGTGCTTTGCATTCTTGGACGAGG-3’ |
| CASP8 | Forward 5’-ATTCCGCAAAGGAAGCAAGAACCC-3’  Reverse 5’-TGCCTGGTGTCTGAAGTTCCCTTT-3’ |
| CTSB | Forward 5’-TATGCCACTGGTTTGCATTGCTGG-3’  Reverse 5’-TGTACCTTGGCAGGACAGTGGAAT-3’ |
| DAPK1 | Forward 5’-AATGGAGTTGGCGATTTCAGCGTG-3’  Reverse 5’-AAGGGACTTCAGGAAACTGAGCCA-3’ |
| GABARAP | HATPL-I |
| HTT | Forward 5’-CAGAACAGCACGGAAAAGTT-3’  Reverse 5’-AACAGTTGCCATCATTGGTT-3 |
| IGF1 | Forward 5’-TGAAGATGCACACCATGTCCTCCT-3’  Reverse 5’-AACTGAAGAGCATCCACCAGCTCA-3’ |
| PINK1 | VHPS-6904 |
| PARKIN | CHPS-1 |
| P62/SQSTM1 | VHPS-8844 |
| SNCA | Forward 5’-ACAAGTGCTCAGTTCCAATGTGCC-3’  Reverse 5’-GTGAAAGGGAAGCACCGAAATGCT-3’ |
| TNF | VHPS-9415 |
| TNFSF10 | Forward 5’-GGACCATGAAGCCAGTTTTT-3’  Reverse 5’-TGGTTGTGGCTGCTCTACTC-3’ |
| TP73 | VHPS-9506 |
| ULK2 | Forward 5’-AGATACGTGCCTTACGGTGCTTCA-3’  Reverse 5’-AGATGGCGTAAGGTGTCTGTGTGT-3’ |

Table S2. List of primers used in autophagy array confirmation.
